# Syntheses and crystal structures of 4-(4-meth­oxy­phen­yl)piperazin-1-ium 4-methyl­benzoate monohydrate and bis­[4-(4-meth­oxy­phen­yl)piperazin-1-ium] benzene-1,2-di­carboxyl­ate

**DOI:** 10.1107/S2056989022008337

**Published:** 2022-08-26

**Authors:** Holehundi J. Shankara Prasad, Devaraju, Vinaya, Hemmige S. Yathirajan, Sean R. Parkin, Christopher Glidewell

**Affiliations:** a Department of Chemistry, Yuvaraja’s College, University of Mysore, Mysore-570 005, India; b Department of Studies in Chemistry, University of Mysore, Manasagangotri, Mysore-570 006, India; c Department of Chemistry, University of Kentucky, Lexington, KY 40506, USA; d School of Chemistry, University of St Andrews, St Andrews, Fife KY16 9ST, UK; Purdue University, USA

**Keywords:** piperazine, co-crystallization, crystal structure, mol­ecular structure, hydrogen bonding, supra­molecular assembly, twinning by inversion

## Abstract

Co-crystallization of *N*-(4-meth­oxy­phen­yl)piperazine with 4-methyl­benzoic acid and with benzene-1,2-di­carb­oxy­lic acid yields the salts 4-(4-meth­oxy­phen­yl)piperazin-1-ium 4-methyl­benzoate monohydrate, C_11_H_17_N_2_O^+^·C_8_H_7_O_2_^−^·H_2_O (I), and bis­[4-(4-meth­oxy­phen­yl)piperazin-1-ium] benzene-1,2-di­carboxyl­ate, 2C_11_H_17_N_2_O^+^·C_8_H_4_O_4_^2−^ (II). These salts both crystallize with *Z*′ = 2, in space groups *P*  and *Pna*2_1_, respectively. In compound (I), a combination of four O—H⋯O, four N—H⋯O, one C—H⋯O and one C—H⋯π(arene) hydrogen bonds link the six independent components into complex sheets, within which the two piperazine rings, the two anions, and the two water mol­ecules are related by an approximate, non-crystallographic translation along the *b*-axis direction. In compound (II), sheets containing *R* ^4^_4_(18) and *R* ^10^_12_(38) rings are formed by the combined action of eight independent N—H⋯O hydrogen bonds. Comparisons are made with the structures of some related compounds.

## Abstract 2

Two salts derived from *N*-(4-meth­oxy­phen­yl)piperazine crystallize with six independent mol­ecular species in the asymmetric unit. In each compound, multiple hydrogen bonds link these components into complex sheets.

## 1. Chemical context

**Figure 1:**
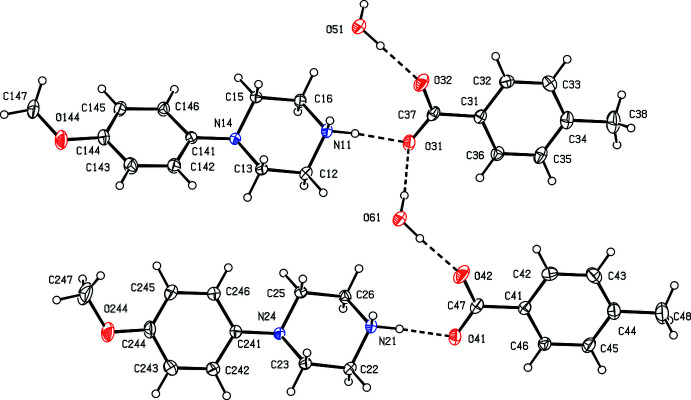
The six independent components in the structure of compound (I), showing the atom-labelling scheme and the hydrogen bonds, drawn as dashed lines, within the selected asymmetric unit. Displacement ellipsoids are drawn at the 30% probability level.

Piperazine derivatives can exhibit a very wide range of biological activity (Asif, 2015 ▸; Brito *et al.*, 2019 ▸). In addition, *N*-(4-meth­oxy­phen­yl)piperazine (MeOPP) is a recreational drug whose action on human physiology resembles that of amphetamines, but which appears to have significantly lower potential for abuse (Nagai *et al.*, 2007 ▸). With these considerations in mind, we have recently initiated a structural study of MeOPP and its derivatives (Kiran Kumar, Yathirajan, Foro *et al.*, 2019 ▸; Kiran Kumar, Yathirajan, Sagar *et al.*, 2019 ▸; Kiran Kumar *et al.*, 2020 ▸): this has included the structures of a number of salts derived from simple aromatic acids (Kiran Kumar, Yathirajan, Foro *et al.*, 2019 ▸; Kiran Kumar *et al.*, 2020 ▸). In a continuation of these earlier studies, we now report the structures of two further salts, namely 4-(4-meth­oxy­phen­yl)piperazin-1-ium 4-methyl­benzoate monohydrate (I) and bis­[4-(4-meth­oxy­phen­yl)piperazin-1-ium] benzene-1,2-di­carboxyl­ate (II) (see scheme and Figs. 1 ▸–3 ▸ ▸).

## 2. Structural commentary

Co-crystallization of *N*-(4-meth­oxy­phen­yl)piperazine and 4-methyl­benzoic acid yielded a 1:1 salt, which crystallized from methanol–ethyl acetate in air as a monohydrate, with *Z*′ = 2 in space group *P* 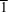 (Fig. 1 ▸). A search for possible additional symmetry revealed none. The possibility of any such symmetry is effectively precluded by the different orientations of the 4-meth­oxy­phenyl unit relative to the piperazine ring in the two independent cations, as indicated by the values of the torsion angles C*x*3—N*x*4—C*x*41—C*x*42 (*x* = 1 or 2, Fig. 1 ▸), −5.45 (18)° when *x* = 1, but −46.92 (17)° when *x* = 2. Apart from this difference, the other pairs of corresponding units (the two piperazine rings, the two anions, and the two water mol­ecules) are related by an approximate, non-crystallographic translation (*x*, 0.5 + *y*, z). Although there are six independent components in the structure, it is possible to select a compact asymmetric unit in which the components are linked by three O—H⋯O hydrogen bonds and two N—H⋯O hydrogen bonds (Fig. 1 ▸, Table 1 ▸). 
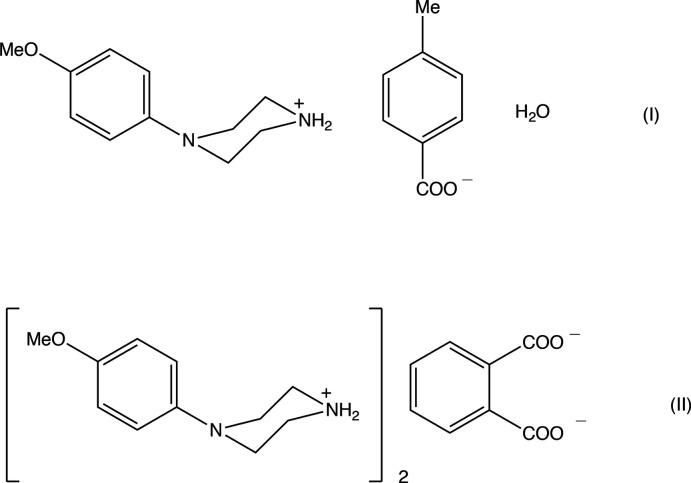


**Figure 2:**
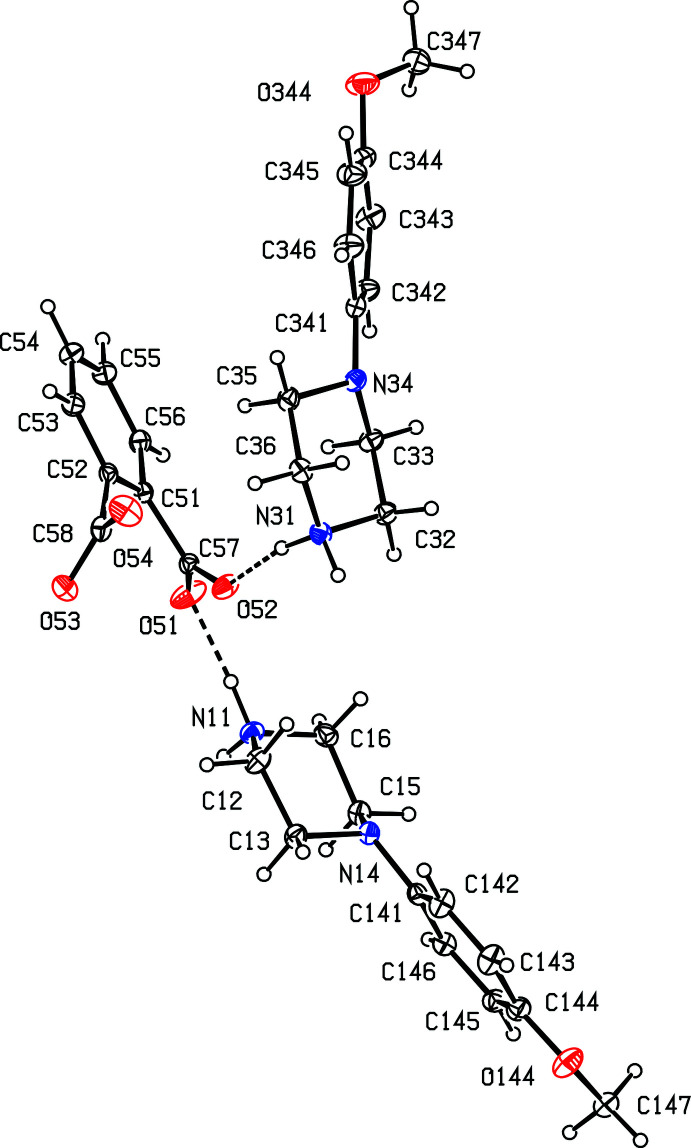
The independent components in the type 1 ion triplet in compound (II), showing the atom-labelling scheme and the hydrogen bonds, drawn as dashed lines, within the selected triplet. Displacement ellipsoids are drawn at the 30% probability level

**Figure 3:**
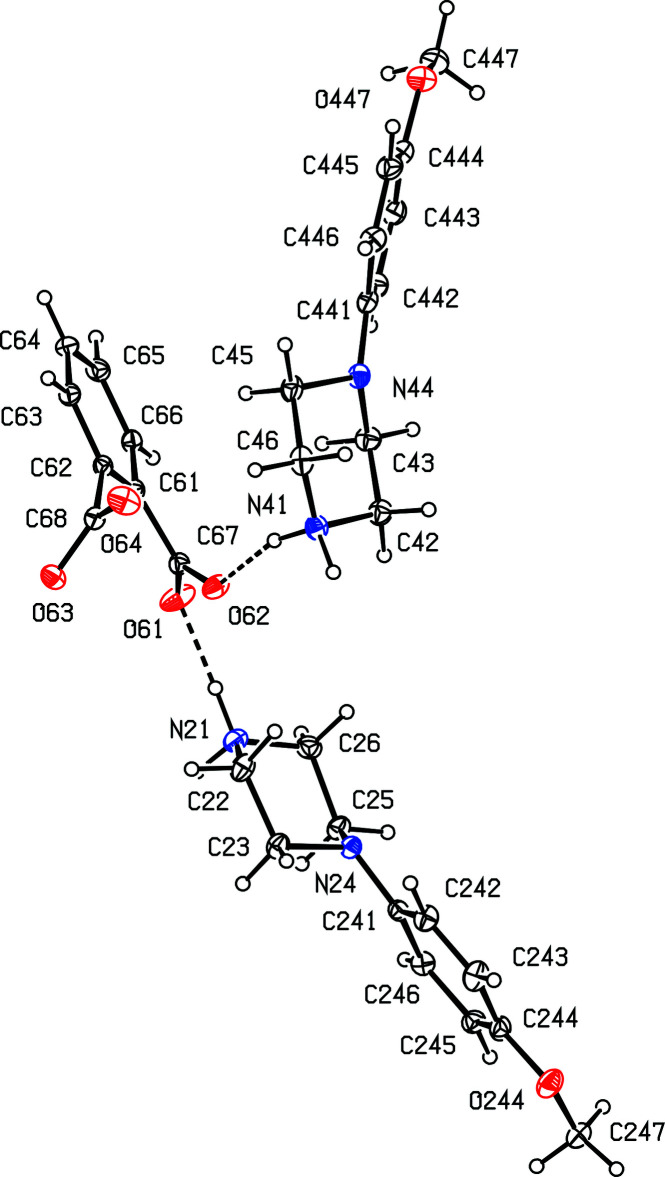
The independent components in the type 2 ion triplet in compound (II), showing the atom-labelling scheme and the hydrogen bonds, drawn as dashed lines, within the selected triplet. Displacement ellipsoids are drawn at the 30% probability level

Compound (II), formed by co-crystallization of *N*-(4-meth­oxy­phen­yl)piperazine with benzene-1,2-di­carb­oxy­lic acid (phthalic acid), is a 2:1 salt that crystallizes in solvent-free form with *Z*′ = 2 in space group *Pna*2_1_. A search for possible additional symmetry revealed none. As for compound (I), there are six independent components in the structure of (II), four cations and two anions, providing a considerable degree of choice in the specification of the asymmetric unit. The selection here consists of two similar ion triplets, each comprising two cations and one anion, which are linked in each triplet by two N—H⋯O hydrogen bonds (Figs. 2 ▸ and 3 ▸). It will be convenient to refer to the ion triplet containing atom N11 (Fig. 2 ▸) as of type 1, and that containing atom N21 (Fig. 3 ▸) as of type 2.

In the cations of compound (I), the meth­oxy C atoms are close to the plane of the adjacent rings, with displacements from these planes of 0.118 (3) and 0.073 (4) Å for atoms C147 and C247, respectively. In compound (II), the corresponding displacements are 0.242 (6), 0.070 (6) and 0.097 (6) Å for atoms C147, C247 and C447, respectively, but 0.750 (6) Å for atom C347. At the same time, the pairs of exocyclic O—C—C angles at C144 and C244 in (I), and at C144, C244 and C444 in (II) all differ by *ca* 10°. This behaviour is characteristic of planar and near-planar alk­oxy­arenes (Seip & Seip, 1973 ▸; Ferguson *et al.*, 1996 ▸; Kiran Kumar *et al.*, 2020 ▸). On the other hand, the difference between the exocyclic angles at atom C344 in (II) is only 6.7 (5)°.

## 3. Supra­molecular features

**Figure 4:**
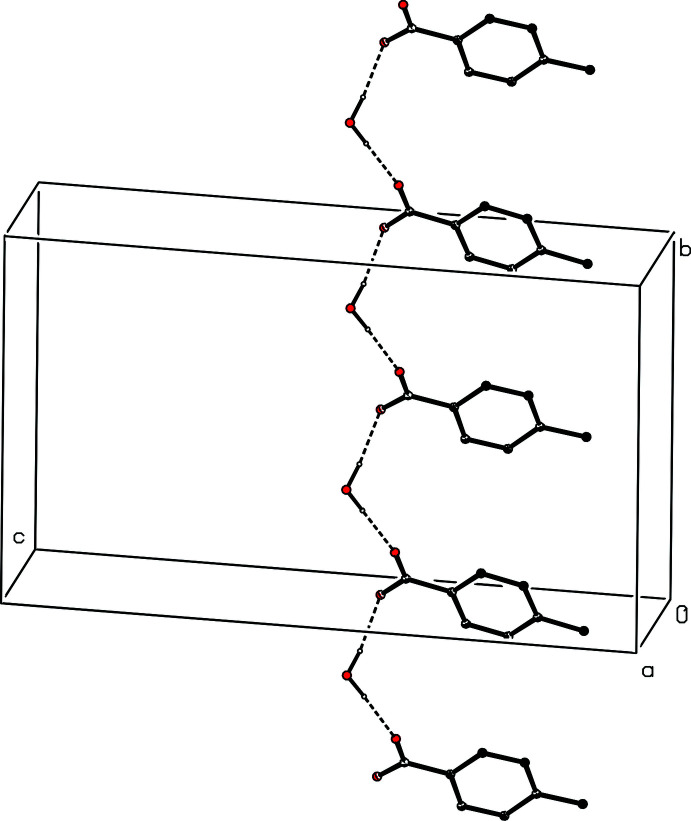
Part of the crystal structure of compound (I) showing the formation of a 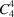 (12) chain of two types of anion and two types of water mol­ecule running parallel to [010]. Hydrogen bonds are drawn as dashed lines and, for the sake of clarity, the cations and the H atoms bonded to C atoms in the anions have been omitted.

**Figure 5:**
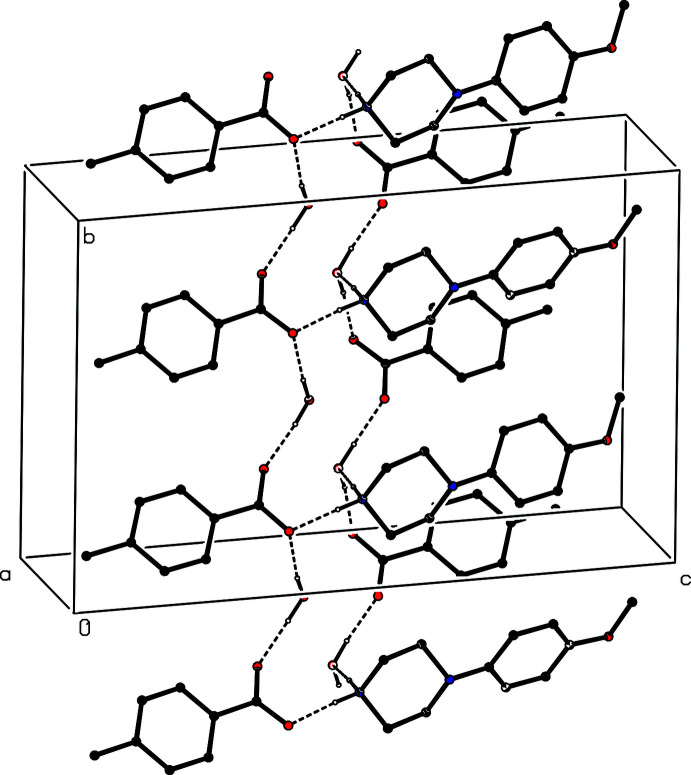
Part of the crystal structure of compound (I) showing the formation of a ribbon of 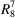 (20) rings running parallel to [010]. Hydrogen bonds are drawn as dashed lines and, for the sake of clarity, the H atoms bonded to C atoms have been omitted.

**Figure 6:**
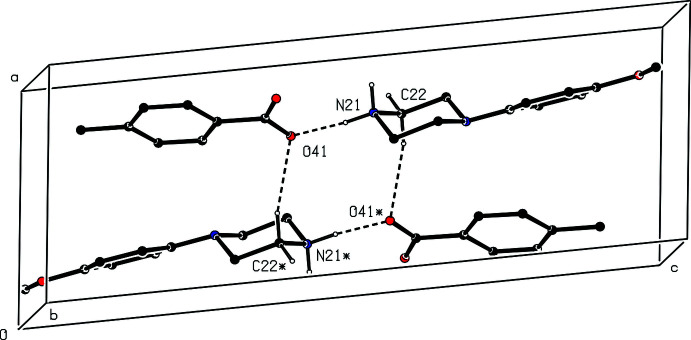
Part of the crystal structure of compound (I) showing the formation of an 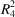 (10) ring linking adjacent [010] chains. Hydrogen bonds are drawn as dashed lines and, for the sake of clarity, the H atoms bonded to those C atoms that are not involved in the motif shown have been omitted. The atoms marked with an asterisk (*) are at the symmetry position (1 − *x*, −*y*, 1 − *z*).

**Figure 7:**
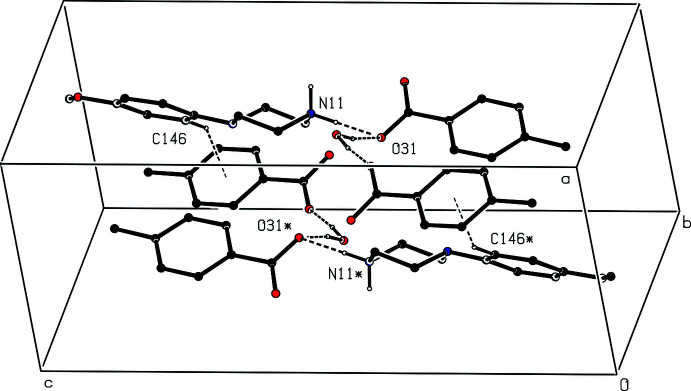
Part of the crystal structure of compound (I) showing the formation of a ring containing O—H⋯O, N—H⋯O and C—H⋯π(arene) hydrogen bonds that link adjacent [010] chains. Hydrogen bonds are drawn as dashed lines and, for the sake of clarity, the H atoms bonded to those C atoms that are not involved in the motif shown have been omitted. The atoms marked with an asterisk (*) are at the symmetry position (1 − *x*, 1 − *y*, 1 − *z*).

The supra­molecular assembly of compound (I) is di-periodic (propagates in two-dimensions) and is built from a combination of O—H⋯O, N—H⋯O, C—H⋯O and C—H⋯π(arene) hydrogen bonds (Table 1 ▸). However, the assembly can readily be analysed in terms of a number of simple substructures (Ferguson *et al.*, 1998*a* ▸,*b* ▸; Gregson *et al.*, 2000 ▸). The two independent anions and the two independent water mol­ecules are linked by O—H⋯O hydrogen bonds to form a 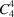 (12) (Etter, 1990 ▸; Etter *et al.*, 1990 ▸; Bernstein *et al.*, 1995 ▸) chain running parallel to the [010] direction (Fig. 4 ▸). Inversion-related pairs of chains of this type are linked by the two types of cation to form a mol­ecular ribbon in the form of a chain of edge-fused 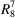 (20) rings parallel to [010] along the line (1, *y*, 0.5) (Fig. 5 ▸). The ribbons along [010] are linked into sheets lying parallel to (001) by a combination of C—H⋯O and C—H⋯π(arene) hydrogen bonds, and it is convenient to consider separately the sub-structures formed by these two types of inter­action. In the simpler of these two sub-structures, (Fig. 6 ▸), inversion-related ion pairs are linked by C—H⋯O hydrogen bonds (Table 1 ▸) to form an 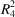 (10) ring, which links the chains along (1, *y*, 0.5) and (0, *y*, 0.5). The second sub-structure (Fig. 7 ▸) contains C—H⋯π(arene) hydrogen bonds and also includes water mol­ecules but, again, it links the chains along (1, *y*, 0.5) and (0, *y*, 0.5). Propagation of these motifs by inversion thus links adjacent [010] chains into a complex sheet lying parallel to (001).

**Figure 8:**
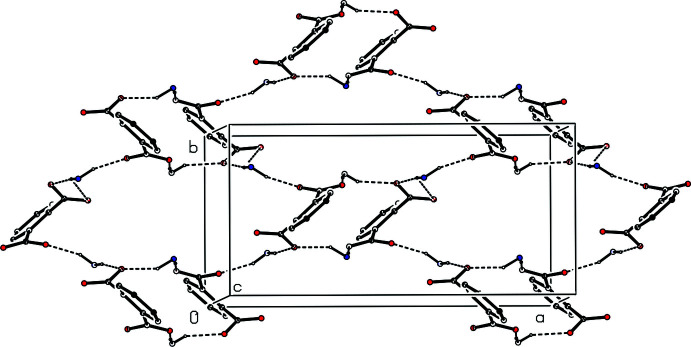
A schematic representation of part of the crystal structure of compound (II) showing the formation of a sheet of 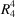 (18) and 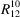 (38) rings. Hydrogen bonds are drawn as dashed lines and, for the sake of clarity, the H atoms of the anions have been omitted and only the NH_2_ groups of the cations are shown.

There are eight independent N—H⋯O hydrogen bonds in the structure of compound (II) (Table 2 ▸). Four of these lie within the two ion triplets that were selected as the asymmetric unit (Figs. 2 ▸ and 3 ▸), and the other four act to link the type 1 and type 2 triplets into sheets of alternating 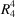 (18) and 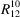 (38) rings lying parallel to (001) (Fig. 8 ▸). Two sheets of this type, which are related to one another by the action of the 2_1_ screw axis, pass through each unit cell, in the domains 0.25 < *z* < 0.75 and 0.75 < *z* < 1.25, but there are no direction-specific inter­actions between adjacent sheets: the C—H⋯O and C—H⋯π(arene) hydrogen bonds all lie within a single sheet.

## 4. Database survey

In addition to the structures of a number of salts formed between *N*-(4-meth­oxy­phen­yl)piperazine and carb­oxy­lic acids (Kiran Kumar, Yathirajan, Foro *et al.*, 2019 ▸; Kiran Kumar, Yathirajan, Sagar *et al.*, 2019 ▸), structures have also been reported for the chloride (Zia-ur-Rehman *et al.*, 2009 ▸) and tetra­(iso­thio­cyanato)­cobaltate(II) salts (Gharbi *et al.*, 2021 ▸). By contrast, the only structures reported for salts of the isomeric *N*-(3-meth­oxy­phen­yl)piperazine are those of the maleate (Verdonk *et al.*, 1997 ▸) and the 4-(3-meth­oxy­phen­yl)piperazin-1-carboxyl­ate (Özdemir, 2021 ▸). In addition to the structures reported for the picrate (Verdonk *et al.*, 1997 ▸) and 6-chloro-5-isopropyl-2,4-dioxo-3,4-di­hydro-2*H*-pyrimidin-1-ide (Al-Omary *et al.*, 2014 ▸) salts derived from *N*-(2-meth­oxy­phen­yl)piperazine, we have recently reported (Harish Chinthal *et al.*, 2020 ▸) the structures of fifteen salts formed by this piperazine with organic acids, where it was found that the supra­molecular assemblies range from finite (non-periodic) aggregates through mono-, di- and tri-periodic arrangements.

## 5. Synthesis and crystallization

For the preparation of compounds (I) and (II), a solution of *N*-(4-meth­oxy­piperazine (100 mg, 0.52 mmol) in methanol (10 ml) was mixed with a solution of the appropriate acid, 4-methyl­benzoic acid (76 mg, 0.52 mmol) for (I), or benzene-1,2-di­carb­oxy­lic acid (phthalic acid, 86 mg, 0.52 mmol) for (II), each in methanol (10 ml). The mixtures were stirred briefly and then set aside at ambient temperature, giving colourless crystals of compounds (I) and (II) after a few days: compound (I), yield 80%, m.p. 413–416 K, compound (II), yield 80%, m.p. 446–447 K. Crystals suitable for single-crystal X-ray diffraction were grown by slow evaporation, at ambient temperature and in the presence of air, of solutions in methanol–ethyl acetate (initial composition 1:1, *v*/*v*).

## 6. Data collection and structure refinement

Crystals of (I) shattered on cooling to 90 K, while those of compound (II) showed faint satellite reflections at 90 K that gradually diminished in intensity on warming. At the data collection temperature of 180–K, crystals of (I) remained intact and the satellite reflections observed for (II) were absent. Crystal data, data collection and refinement details are summarized in Table 3 ▸. All H atoms were located in difference maps. The H atoms bonded to C atoms were then treated as riding atoms in geometrically idealised positions with C—H distances 0.95 Å (aromatic), 0.98 Å (CH_3_ or 0.99 Å (CH_2_), and with *U*_iso_(H) = *kU*_eq_(C), where *k* = 1.5 for the methyl groups, which were permitted to rotate but not to tilt, and 1.2 for all other H atoms bonded to C atoms. For the H atoms bonded to N or O atoms, the atomic coordinates were refined with *U*_iso_(H) = 1.2*U*_eq_(N) or 1.5*U*_eq_(O), giving the N—H and O—H distances shown in Tables 1 ▸ and 2 ▸. For compound (II), Cu *Kα* radiation was used to facilitate establishing a unique orientation for the structure with respect to the polar axis direction. For the crystal selected for data collection, however, the value of the Flack *x* parameter (Flack, 1983 ▸), obtained in the conventional way via full-matrix least-squares refinement, *i.e. x* = 0.45 (18) was inconclusive due to its high standard uncertainty, while that calculated using 4511 quotients (Parsons *et al.*, 2013 ▸) of the type [(*I* ^+^) − (*I* ^−^)]/[(*I* ^+^) + (*I* ^−^)] was *x* = 0.45 (5), strongly suggesting the likelihood of twinning by inversion.

## Supplementary Material

Crystal structure: contains datablock(s) global, I, II. DOI: 10.1107/S2056989022008337/zl5034sup1.cif

Structure factors: contains datablock(s) I. DOI: 10.1107/S2056989022008337/zl5034Isup2.hkl

Structure factors: contains datablock(s) II. DOI: 10.1107/S2056989022008337/zl5034IIsup3.hkl

Supporting information file. DOI: 10.1107/S2056989022008337/zl5034Isup4.cml

Supporting information file. DOI: 10.1107/S2056989022008337/zl5034IIsup5.cml

CCDC references: 2202591, 2202590

Additional supporting information:  crystallographic information; 3D view; checkCIF report

## Figures and Tables

**Table 1:** Hydrogen-bond geometry (Å, °) for (I) *Cg*1 and *Cg*2 represent the centroids of the C31–C36 and C41–C46 rings, respectively.

*D*—H⋯*A*	*D*—H	H⋯*A*	*D*⋯*A*	*D*—H⋯*A*
N11—H11⋯O31	0.946 (16)	1.793 (16)	2.7366 (16)	174.5 (13)
N11—H12⋯O61^i^	0.917 (15)	1.891 (15)	2.7969 (16)	169.2 (13)
N21—H21⋯O41	0.949 (16)	1.813 (16)	2.7592 (16)	174.1 (14)
N21—H22⋯O51^i^	0.904 (15)	1.909 (15)	2.8047 (16)	170.9 (13)
O51—H51⋯O41^ii^	0.89 (2)	1.89 (2)	2.7712 (16)	170.0 (18)
O51—H52⋯O32	0.918 (18)	1.739 (18)	2.6539 (16)	174.3 (15)
O61—H61⋯O42	0.906 (19)	1.733 (19)	2.6315 (16)	171.2 (15)
O61—H62⋯O31	0.92 (2)	1.85 (2)	2.7593 (15)	169.1 (19)
C22—H22*B*⋯O41^iii^	0.99	2.56	3.5266 (19)	167
C142—H142⋯*Cg*1^i^	0.95	2.83	3.5976 (16)	138
C146—H146⋯*Cg*2^iv^	0.95	2.73	3.5478 (16)	145

Symmetry codes: (i) 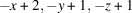 ; (ii) 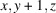 ; (iii) 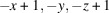 ; (iv) 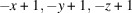 .

**Table 2:** Hydrogen-bond geometry (Å, °) for (II) *Cg*3 and *Cg*4 represent the centroids of the C61–C66 and C51–C56 rings, respectively.

*D*—H⋯*A*	*D*—H	H⋯*A*	*D*⋯*A*	*D*—H⋯*A*
N11—H11⋯O51	0.99 (4)	1.73 (4)	2.714 (4)	175 (2)
N11—H12⋯O63^i^	0.80 (4)	2.03 (4)	2.738 (3)	148 (3)
N21—H21⋯O61	0.99 (4)	1.72 (4)	2.707 (4)	177 (3)
N21—H22⋯O53^ii^	1.01 (4)	1.88 (4)	2.744 (3)	142 (3)
N31—H31⋯O63^iii^	0.94 (3)	1.77 (3)	2.685 (3)	163 (3)
N31—H32⋯O52	0.92 (4)	1.83 (4)	2.732 (3)	164 (3)
N41—H41⋯O53^iv^	1.00 (4)	1.74 (4)	2.711 (3)	163 (3)
N41—H42⋯O62	0.90 (4)	1.89 (4)	2.740 (3)	157 (3)
C36—H36*A*⋯O51^v^	0.99	2.40	3.354 (4)	160
C46—H46*A*⋯O61^v^	0.99	2.42	3.359 (4)	158
C13—H13*B*⋯*Cg*3^i^	0.99	2.84	3.795 (3)	161
C23—H23*B*⋯*Cg*4^i^	0.99	2.85	3.800 (3)	162

Symmetry codes: (i) 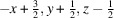 ; (ii) 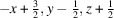 ; (iii) 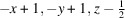 ; (iv) 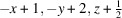 ; (v) 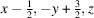 .

**Table 3:** Experimental details

	(I)	(II)
Crystal data		
Chemical formula	C_11_H_17_N_2_O^+^·C_8_H_7_O_2_^−^·H_2_O	2C_11_H_17_N_2_O^+^·C_8_H_4_O_4_^2−^
*M*_r_	346.42	550.64
Crystal system, space group	Triclinic, *P* 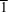	Orthorhombic, *P* *n* *a*2_1_
Temperature (K)	180	180
*a*, *b*, *c* (Å)	7.4141 (5), 12.3595 (11), 19.9917 (17)	17.8424 (4), 8.8124 (2), 34.9337 (9)
α, β, γ (°)	86.695 (2), 83.654 (2), 82.182 (3)	90, 90, 90
*V* (Å^3^)	1802.1 (3)	5492.8 (2)
*Z*	4	8
Radiation type	Mo *K*α	Cu *K*α
μ (mm^−1^)	0.09	0.76
Crystal size (mm)	0.30 × 0.18 × 0.08	0.26 × 0.24 × 0.04

Data collection		
Diffractometer	Bruker D8 Venture dual source	Bruker D8 Venture dual source
Absorption correction	Multi-scan (*SADABS*; Krause *et al.*, 2015 ▸)	Multi-scan (*SADABS*; Krause *et al.*, 2015 ▸)
*T*_min_, *T*_max_	0.938, 0.971	0.814, 0.942
No. of measured, independent and observed [*I* > 2σ(*I*)] reflections	54020, 8288, 5772	36488, 10053, 9727
*R*_int_	0.050	0.031
(sin θ/λ)_max_ (Å^−1^)	0.650	0.610

Refinement		
*R*[*F* ^2^ > 2σ(*F* ^2^)], *wR*(*F* ^2^), *S*	0.046, 0.132, 1.04	0.037, 0.098, 1.07
No. of reflections	8288	10053
No. of parameters	479	750
No. of restraints	0	1
H-atom treatment	H atoms treated by a mixture of independent and constrained refinement	H atoms treated by a mixture of independent and constrained refinement
Δρ_max_, Δρ_min_ (e Å^−3^)	0.30, −0.18	0.27, −0.17
Absolute structure	–	Flack *x* determined using 4511 quotients [(*I* ^+^)−(*I* ^−^)]/[(*I* ^+^)+(*I* ^−^)] (Parsons *et al.*, 2013 ▸)
Absolute structure parameter	–	0.45 (5)

Computer programs: *APEX3* (Bruker, 2016 ▸), *SHELXT2014/5* (Sheldrick, 2015*a* ▸), *SHELXL2014* (Sheldrick, 2015*b* ▸) and *PLATON* (Spek, 2020 ▸).

## supplementary crystallographic information

### 4-(4-Methoxyphenyl)piperazin-1-ium 4-methylbenzoate monohydrate (I) Crystal data

**Table:**

C_11_H_17_N_2_O^+^·C_8_H_7_O_2_^−^·H_2_O	*Z* = 4
*M**_r_* = 346.42	*F*(000) = 744
Triclinic, *P*1	*D*_x_ = 1.277 Mg m^−^^3^
*a* = 7.4141 (5) Å	Mo *K*α radiation, λ = 0.71073 Å
*b* = 12.3595 (11) Å	Cell parameters from 8258 reflections
*c* = 19.9917 (17) Å	θ = 1.9–27.5°
α = 86.695 (2)°	µ = 0.09 mm^−^^1^
β = 83.654 (2)°	*T* = 180 K
γ = 82.182 (3)°	Needle, colourless
*V* = 1802.1 (3) Å^3^	0.30 × 0.18 × 0.08 mm

### 4-(4-Methoxyphenyl)piperazin-1-ium 4-methylbenzoate monohydrate (I) Data collection

**Table:**

Bruker D8 Venture dual source diffractometer	8288 independent reflections
Radiation source: microsource	5772 reflections with *I* > 2σ(*I*)
Multilayer optics monochromator	*R*_int_ = 0.050
φ and ω scans	θ_max_ = 27.5°, θ_min_ = 1.9°
Absorption correction: multi-scan (SADABS; Krause *et al.*, 2015)	*h* = −9→8
*T*_min_ = 0.938, *T*_max_ = 0.971	*k* = −16→16
54020 measured reflections	*l* = −25→25

### 4-(4-Methoxyphenyl)piperazin-1-ium 4-methylbenzoate monohydrate (I) Refinement

**Table:**

Refinement on *F*^2^	Primary atom site location: dual
Least-squares matrix: full	Hydrogen site location: mixed
*R*[*F*^2^ > 2σ(*F*^2^)] = 0.046	H atoms treated by a mixture of independent and constrained refinement
*wR*(*F*^2^) = 0.132	*w* = 1/[σ^2^(*F*_o_^2^) + (0.0586*P*)^2^ + 0.3838*P*] where *P* = (*F*_o_^2^ + 2*F*_c_^2^)/3
*S* = 1.04	(Δ/σ)_max_ = 0.001
8288 reflections	Δρ_max_ = 0.30 e Å^−^^3^
479 parameters	Δρ_min_ = −0.18 e Å^−^^3^
0 restraints	

### 4-(4-Methoxyphenyl)piperazin-1-ium 4-methylbenzoate monohydrate (I) Special details

**Table:**

Geometry. All esds (except the esd in the dihedral angle between two l.s. planes) are estimated using the full covariance matrix. The cell esds are taken into account individually in the estimation of esds in distances, angles and torsion angles; correlations between esds in cell parameters are only used when they are defined by crystal symmetry. An approximate (isotropic) treatment of cell esds is used for estimating esds involving l.s. planes.

### 4-(4-Methoxyphenyl)piperazin-1-ium 4-methylbenzoate monohydrate (I) Fractional atomic coordinates and isotropic or equivalent isotropic displacement parameters (Å^2^)

**Table:**

	*x*	*y*	*z*	*U*_iso_*/*U*_eq_	
N11	0.76096 (16)	0.62068 (10)	0.54566 (6)	0.0267 (3)	
H11	0.733 (2)	0.6044 (12)	0.5026 (8)	0.032*	
H12	0.880 (2)	0.6350 (12)	0.5392 (7)	0.032*	
C12	0.7518 (2)	0.52384 (11)	0.59281 (7)	0.0310 (3)	
H12A	0.8359	0.4610	0.5742	0.037*	
H12B	0.6260	0.5039	0.5981	0.037*	
C13	0.8047 (2)	0.54855 (11)	0.66075 (7)	0.0294 (3)	
H13A	0.7922	0.4845	0.6922	0.035*	
H13B	0.9346	0.5612	0.6559	0.035*	
N14	0.69096 (14)	0.64449 (9)	0.68905 (5)	0.0240 (2)	
C15	0.69514 (18)	0.74004 (10)	0.64205 (6)	0.0255 (3)	
H15A	0.8203	0.7610	0.6359	0.031*	
H15B	0.6112	0.8024	0.6614	0.031*	
C16	0.63897 (18)	0.71658 (11)	0.57442 (6)	0.0280 (3)	
H16A	0.5105	0.7011	0.5798	0.034*	
H16B	0.6472	0.7814	0.5433	0.034*	
C141	0.71549 (17)	0.66404 (11)	0.75664 (6)	0.0243 (3)	
C142	0.8279 (2)	0.59160 (12)	0.79520 (7)	0.0355 (3)	
H142	0.8979	0.5294	0.7751	0.043*	
C143	0.8388 (2)	0.60898 (14)	0.86214 (7)	0.0426 (4)	
H143	0.9144	0.5578	0.8876	0.051*	
C144	0.7416 (2)	0.69941 (13)	0.89278 (7)	0.0369 (3)	
C145	0.6318 (2)	0.77281 (12)	0.85531 (7)	0.0348 (3)	
H145	0.5649	0.8358	0.8755	0.042*	
C146	0.61891 (19)	0.75492 (12)	0.78825 (7)	0.0299 (3)	
H146	0.5421	0.8061	0.7632	0.036*	
O144	0.76486 (19)	0.70874 (10)	0.95950 (5)	0.0570 (4)	
C147	0.6805 (3)	0.80445 (16)	0.99074 (8)	0.0634 (6)	
H17A	0.7279	0.8681	0.9672	0.095*	
H17B	0.7069	0.8009	1.0378	0.095*	
H17C	0.5479	0.8111	0.9889	0.095*	
N21	0.75064 (16)	0.11429 (10)	0.54577 (6)	0.0264 (2)	
H21	0.723 (2)	0.0975 (12)	0.5026 (8)	0.032*	
H22	0.867 (2)	0.1300 (12)	0.5412 (7)	0.032*	
C22	0.7444 (2)	0.01754 (11)	0.59339 (7)	0.0299 (3)	
H22A	0.8321	−0.0443	0.5755	0.036*	
H22B	0.6202	−0.0048	0.5982	0.036*	
C23	0.79276 (19)	0.04501 (11)	0.66152 (7)	0.0291 (3)	
H23A	0.7829	−0.0189	0.6933	0.035*	
H23B	0.9212	0.0604	0.6571	0.035*	
N24	0.67239 (14)	0.14015 (9)	0.68904 (5)	0.0239 (2)	
C25	0.67558 (18)	0.23465 (11)	0.64143 (6)	0.0257 (3)	
H25A	0.7999	0.2569	0.6356	0.031*	
H25B	0.5892	0.2967	0.6599	0.031*	
C26	0.62318 (18)	0.20845 (11)	0.57381 (6)	0.0274 (3)	
H26A	0.4963	0.1903	0.5790	0.033*	
H26B	0.6283	0.2731	0.5424	0.033*	
C241	0.70163 (17)	0.16099 (11)	0.75612 (6)	0.0249 (3)	
C242	0.7146 (2)	0.07628 (13)	0.80489 (7)	0.0428 (4)	
H242	0.7053	0.0041	0.7929	0.051*	
C243	0.7406 (3)	0.09470 (14)	0.87012 (8)	0.0518 (5)	
H243	0.7485	0.0352	0.9024	0.062*	
C244	0.7554 (2)	0.19800 (14)	0.88941 (7)	0.0391 (4)	
C245	0.7391 (2)	0.28321 (14)	0.84304 (8)	0.0441 (4)	
H245	0.7458	0.3553	0.8557	0.053*	
C246	0.7126 (2)	0.26424 (13)	0.77696 (7)	0.0402 (4)	
H246	0.7018	0.3244	0.7452	0.048*	
O244	0.7874 (2)	0.20623 (11)	0.95543 (6)	0.0610 (4)	
C247	0.8036 (3)	0.31074 (18)	0.97722 (10)	0.0723 (7)	
H27A	0.9015	0.3418	0.9486	0.109*	
H27B	0.8328	0.3045	1.0240	0.109*	
H27C	0.6877	0.3586	0.9742	0.109*	
C31	0.75414 (17)	0.59237 (11)	0.30344 (7)	0.0263 (3)	
C32	0.8027 (2)	0.66491 (12)	0.25084 (7)	0.0354 (3)	
H32	0.8517	0.7290	0.2600	0.042*	
C33	0.7799 (2)	0.64400 (14)	0.18531 (8)	0.0437 (4)	
H33	0.8126	0.6946	0.1499	0.052*	
C34	0.7104 (2)	0.55090 (14)	0.17012 (8)	0.0399 (4)	
C35	0.6671 (2)	0.47747 (13)	0.22262 (7)	0.0375 (4)	
H35	0.6231	0.4119	0.2132	0.045*	
C36	0.68710 (18)	0.49820 (12)	0.28865 (7)	0.0308 (3)	
H36	0.6546	0.4475	0.3240	0.037*	
C37	0.77205 (18)	0.61793 (11)	0.37504 (7)	0.0289 (3)	
O31	0.69445 (14)	0.56144 (8)	0.42179 (5)	0.0355 (2)	
O32	0.86049 (16)	0.69333 (10)	0.38415 (6)	0.0465 (3)	
C38	0.6821 (3)	0.52858 (18)	0.09872 (9)	0.0619 (5)	
H38A	0.6373	0.5972	0.0753	0.093*	
H38B	0.5922	0.4772	0.0995	0.093*	
H38C	0.7986	0.4969	0.0751	0.093*	
C41	0.78088 (17)	0.09275 (11)	0.30254 (7)	0.0257 (3)	
C42	0.85635 (19)	0.16165 (12)	0.25294 (8)	0.0348 (3)	
H42	0.9018	0.2252	0.2652	0.042*	
C43	0.8655 (2)	0.13794 (14)	0.18548 (8)	0.0433 (4)	
H43	0.9165	0.1860	0.1520	0.052*	
C44	0.8020 (2)	0.04568 (14)	0.16608 (7)	0.0409 (4)	
C45	0.7276 (2)	−0.02308 (13)	0.21581 (7)	0.0363 (3)	
H45	0.6831	−0.0868	0.2034	0.044*	
C46	0.71718 (18)	−0.00019 (11)	0.28337 (7)	0.0290 (3)	
H46	0.6661	−0.0484	0.3167	0.035*	
C47	0.77169 (18)	0.11822 (12)	0.37561 (7)	0.0297 (3)	
O41	0.69295 (14)	0.05664 (9)	0.41930 (5)	0.0358 (2)	
O42	0.84243 (17)	0.19826 (10)	0.38947 (6)	0.0529 (3)	
C48	0.8102 (3)	0.01966 (18)	0.09276 (8)	0.0642 (6)	
H48A	0.8524	0.0805	0.0644	0.096*	
H48B	0.6880	0.0087	0.0823	0.096*	
H48C	0.8954	−0.0471	0.0840	0.096*	
O51	0.87815 (14)	0.85863 (9)	0.46119 (5)	0.0333 (2)	
H51	0.813 (3)	0.9175 (16)	0.4445 (9)	0.050*	
H52	0.864 (2)	0.8028 (15)	0.4347 (9)	0.050*	
O61	0.86471 (14)	0.36156 (9)	0.46571 (5)	0.0340 (2)	
H61	0.845 (2)	0.3057 (16)	0.4412 (9)	0.051*	
H62	0.797 (3)	0.4234 (16)	0.4492 (9)	0.051*	

### 4-(4-Methoxyphenyl)piperazin-1-ium 4-methylbenzoate monohydrate (I) Atomic displacement parameters (Å^2^)

**Table:**

	*U*^11^	*U*^22^	*U*^33^	*U*^12^	*U*^13^	*U*^23^
N11	0.0281 (6)	0.0315 (7)	0.0209 (6)	−0.0048 (5)	−0.0022 (4)	−0.0048 (5)
C12	0.0388 (8)	0.0254 (7)	0.0290 (7)	−0.0034 (6)	−0.0017 (6)	−0.0065 (6)
C13	0.0397 (8)	0.0217 (7)	0.0254 (7)	0.0019 (6)	−0.0039 (6)	−0.0014 (5)
N14	0.0294 (6)	0.0214 (6)	0.0205 (5)	−0.0016 (4)	−0.0019 (4)	−0.0019 (4)
C15	0.0307 (7)	0.0219 (7)	0.0230 (6)	−0.0013 (5)	−0.0019 (5)	−0.0004 (5)
C16	0.0310 (7)	0.0281 (7)	0.0239 (7)	0.0009 (5)	−0.0034 (5)	−0.0023 (5)
C141	0.0263 (6)	0.0253 (7)	0.0217 (6)	−0.0048 (5)	−0.0027 (5)	0.0000 (5)
C142	0.0426 (8)	0.0326 (8)	0.0292 (7)	0.0058 (6)	−0.0071 (6)	−0.0023 (6)
C143	0.0540 (10)	0.0418 (9)	0.0303 (8)	0.0071 (7)	−0.0144 (7)	0.0008 (7)
C144	0.0487 (9)	0.0404 (9)	0.0228 (7)	−0.0058 (7)	−0.0085 (6)	−0.0030 (6)
C145	0.0422 (8)	0.0335 (8)	0.0278 (7)	0.0012 (6)	−0.0041 (6)	−0.0074 (6)
C146	0.0340 (7)	0.0308 (8)	0.0244 (7)	0.0021 (6)	−0.0070 (5)	−0.0024 (6)
O144	0.0881 (10)	0.0560 (8)	0.0260 (6)	0.0075 (7)	−0.0207 (6)	−0.0079 (5)
C147	0.1103 (17)	0.0533 (12)	0.0268 (9)	−0.0018 (11)	−0.0142 (9)	−0.0118 (8)
N21	0.0262 (6)	0.0316 (7)	0.0220 (6)	−0.0038 (5)	−0.0028 (4)	−0.0047 (5)
C22	0.0371 (7)	0.0245 (7)	0.0286 (7)	−0.0034 (6)	−0.0038 (6)	−0.0056 (6)
C23	0.0362 (7)	0.0241 (7)	0.0259 (7)	0.0017 (6)	−0.0045 (5)	−0.0018 (5)
N24	0.0274 (6)	0.0227 (6)	0.0213 (5)	−0.0019 (4)	−0.0034 (4)	−0.0015 (4)
C25	0.0276 (7)	0.0228 (7)	0.0255 (7)	0.0001 (5)	−0.0018 (5)	−0.0012 (5)
C26	0.0283 (7)	0.0295 (7)	0.0234 (7)	0.0007 (5)	−0.0039 (5)	−0.0005 (5)
C241	0.0238 (6)	0.0274 (7)	0.0236 (6)	−0.0035 (5)	−0.0021 (5)	−0.0022 (5)
C242	0.0697 (11)	0.0306 (8)	0.0307 (8)	−0.0103 (8)	−0.0128 (7)	0.0008 (6)
C243	0.0867 (14)	0.0402 (10)	0.0300 (8)	−0.0060 (9)	−0.0191 (8)	0.0056 (7)
C244	0.0463 (9)	0.0474 (10)	0.0239 (7)	−0.0008 (7)	−0.0087 (6)	−0.0063 (7)
C245	0.0665 (11)	0.0359 (9)	0.0320 (8)	−0.0076 (8)	−0.0092 (7)	−0.0095 (7)
C246	0.0646 (11)	0.0289 (8)	0.0281 (7)	−0.0080 (7)	−0.0068 (7)	−0.0008 (6)
O244	0.0957 (10)	0.0601 (8)	0.0294 (6)	0.0005 (7)	−0.0246 (6)	−0.0107 (6)
C247	0.1032 (18)	0.0722 (15)	0.0462 (11)	0.0022 (12)	−0.0323 (11)	−0.0265 (10)
C31	0.0226 (6)	0.0281 (7)	0.0281 (7)	0.0011 (5)	−0.0045 (5)	−0.0063 (6)
C32	0.0431 (8)	0.0276 (8)	0.0362 (8)	−0.0041 (6)	−0.0080 (6)	−0.0014 (6)
C33	0.0572 (10)	0.0415 (10)	0.0323 (8)	−0.0056 (8)	−0.0089 (7)	0.0047 (7)
C34	0.0397 (8)	0.0495 (10)	0.0304 (8)	−0.0005 (7)	−0.0072 (6)	−0.0083 (7)
C35	0.0356 (8)	0.0440 (9)	0.0359 (8)	−0.0108 (7)	−0.0032 (6)	−0.0160 (7)
C36	0.0291 (7)	0.0332 (8)	0.0306 (7)	−0.0068 (6)	0.0004 (5)	−0.0062 (6)
C37	0.0262 (7)	0.0286 (7)	0.0314 (7)	0.0050 (6)	−0.0075 (5)	−0.0097 (6)
O31	0.0410 (6)	0.0402 (6)	0.0255 (5)	−0.0021 (5)	−0.0049 (4)	−0.0069 (4)
O32	0.0521 (7)	0.0477 (7)	0.0452 (7)	−0.0166 (5)	−0.0084 (5)	−0.0191 (5)
C38	0.0717 (13)	0.0817 (15)	0.0349 (9)	−0.0065 (11)	−0.0154 (9)	−0.0130 (9)
C41	0.0228 (6)	0.0271 (7)	0.0277 (7)	−0.0004 (5)	−0.0065 (5)	−0.0046 (5)
C42	0.0324 (7)	0.0299 (8)	0.0433 (9)	−0.0060 (6)	−0.0060 (6)	−0.0012 (6)
C43	0.0465 (9)	0.0445 (10)	0.0359 (8)	−0.0040 (7)	0.0017 (7)	0.0074 (7)
C44	0.0474 (9)	0.0446 (9)	0.0282 (8)	0.0061 (7)	−0.0055 (6)	−0.0039 (7)
C45	0.0446 (9)	0.0319 (8)	0.0336 (8)	−0.0004 (6)	−0.0119 (6)	−0.0094 (6)
C46	0.0314 (7)	0.0281 (7)	0.0280 (7)	−0.0029 (6)	−0.0059 (5)	−0.0023 (6)
C47	0.0252 (7)	0.0313 (8)	0.0331 (7)	0.0031 (6)	−0.0090 (5)	−0.0107 (6)
O41	0.0398 (6)	0.0417 (6)	0.0260 (5)	−0.0015 (5)	−0.0063 (4)	−0.0062 (4)
O42	0.0631 (8)	0.0510 (7)	0.0516 (7)	−0.0211 (6)	−0.0090 (6)	−0.0239 (6)
C48	0.0876 (15)	0.0710 (14)	0.0296 (9)	0.0081 (11)	−0.0063 (9)	−0.0081 (9)
O51	0.0338 (5)	0.0334 (6)	0.0341 (6)	−0.0057 (4)	−0.0042 (4)	−0.0090 (5)
O61	0.0363 (6)	0.0333 (6)	0.0340 (6)	−0.0066 (5)	−0.0034 (4)	−0.0104 (5)

### 4-(4-Methoxyphenyl)piperazin-1-ium 4-methylbenzoate monohydrate (I) Geometric parameters (Å, º)

**Table:**

N11—C12	1.4845 (18)	C242—C243	1.375 (2)
N11—C16	1.4917 (17)	C242—H242	0.9500
N11—H11	0.948 (16)	C243—C244	1.377 (2)
N11—H12	0.918 (16)	C243—H243	0.9500
C12—C13	1.5131 (18)	C244—C245	1.364 (2)
C12—H12A	0.9900	C244—O244	1.3784 (17)
C12—H12B	0.9900	C245—C246	1.394 (2)
C13—N14	1.4617 (16)	C245—H245	0.9500
C13—H13A	0.9900	C246—H246	0.9500
C13—H13B	0.9900	O244—C247	1.411 (2)
N14—C141	1.4216 (16)	C247—H27A	0.9800
N14—C15	1.4677 (16)	C247—H27B	0.9800
C15—C16	1.5128 (18)	C247—H27C	0.9800
C15—H15A	0.9900	C31—C36	1.3852 (19)
C15—H15B	0.9900	C31—C32	1.391 (2)
C16—H16A	0.9900	C31—C37	1.5072 (18)
C16—H16B	0.9900	C32—C33	1.383 (2)
C141—C146	1.3917 (18)	C32—H32	0.9500
C141—C142	1.3963 (19)	C33—C34	1.385 (2)
C142—C143	1.380 (2)	C33—H33	0.9500
C142—H142	0.9500	C34—C35	1.387 (2)
C143—C144	1.381 (2)	C34—C38	1.511 (2)
C143—H143	0.9500	C35—C36	1.3865 (19)
C144—O144	1.3771 (17)	C35—H35	0.9500
C144—C145	1.379 (2)	C36—H36	0.9500
C145—C146	1.3870 (19)	C37—O32	1.2424 (17)
C145—H145	0.9500	C37—O31	1.2678 (17)
C146—H146	0.9500	C38—H38A	0.9800
O144—C147	1.406 (2)	C38—H38B	0.9800
C147—H17A	0.9800	C38—H38C	0.9800
C147—H17B	0.9800	C41—C46	1.3885 (19)
C147—H17C	0.9800	C41—C42	1.390 (2)
N21—C22	1.4874 (18)	C41—C47	1.5043 (18)
N21—C26	1.4902 (17)	C42—C43	1.389 (2)
N21—H21	0.950 (16)	C42—H42	0.9500
N21—H22	0.905 (16)	C43—C44	1.382 (2)
C22—C23	1.5144 (18)	C43—H43	0.9500
C22—H22A	0.9900	C44—C45	1.387 (2)
C22—H22B	0.9900	C44—C48	1.512 (2)
C23—N24	1.4706 (16)	C45—C46	1.3876 (19)
C23—H23A	0.9900	C45—H45	0.9500
C23—H23B	0.9900	C46—H46	0.9500
N24—C241	1.4251 (16)	C47—O42	1.2392 (17)
N24—C25	1.4646 (16)	C47—O41	1.2705 (18)
C25—C26	1.5113 (18)	C48—H48A	0.9800
C25—H25A	0.9900	C48—H48B	0.9800
C25—H25B	0.9900	C48—H48C	0.9800
C26—H26A	0.9900	O51—H51	0.89 (2)
C26—H26B	0.9900	O51—H52	0.918 (19)
C241—C246	1.381 (2)	O61—H61	0.904 (19)
C241—C242	1.390 (2)	O61—H62	0.92 (2)
			
C12—N11—C16	109.88 (10)	N21—C26—H26A	109.7
C12—N11—H11	110.3 (9)	C25—C26—H26A	109.7
C16—N11—H11	112.7 (9)	N21—C26—H26B	109.7
C12—N11—H12	108.3 (10)	C25—C26—H26B	109.7
C16—N11—H12	110.4 (9)	H26A—C26—H26B	108.2
H11—N11—H12	105.1 (13)	C246—C241—C242	116.53 (13)
N11—C12—C13	110.18 (11)	C246—C241—N24	123.04 (12)
N11—C12—H12A	109.6	C242—C241—N24	120.40 (12)
C13—C12—H12A	109.6	C243—C242—C241	121.40 (15)
N11—C12—H12B	109.6	C243—C242—H242	119.3
C13—C12—H12B	109.6	C241—C242—H242	119.3
H12A—C12—H12B	108.1	C242—C243—C244	121.06 (15)
N14—C13—C12	112.02 (11)	C242—C243—H243	119.5
N14—C13—H13A	109.2	C244—C243—H243	119.5
C12—C13—H13A	109.2	C245—C244—C243	118.89 (14)
N14—C13—H13B	109.2	C245—C244—O244	125.06 (15)
C12—C13—H13B	109.2	C243—C244—O244	116.05 (14)
H13A—C13—H13B	107.9	C244—C245—C246	119.87 (15)
C141—N14—C13	115.21 (10)	C244—C245—H245	120.1
C141—N14—C15	115.05 (10)	C246—C245—H245	120.1
C13—N14—C15	111.21 (10)	C241—C246—C245	122.22 (14)
N14—C15—C16	111.37 (11)	C241—C246—H246	118.9
N14—C15—H15A	109.4	C245—C246—H246	118.9
C16—C15—H15A	109.4	C244—O244—C247	117.82 (14)
N14—C15—H15B	109.4	O244—C247—H27A	109.5
C16—C15—H15B	109.4	O244—C247—H27B	109.5
H15A—C15—H15B	108.0	H27A—C247—H27B	109.5
N11—C16—C15	109.93 (11)	O244—C247—H27C	109.5
N11—C16—H16A	109.7	H27A—C247—H27C	109.5
C15—C16—H16A	109.7	H27B—C247—H27C	109.5
N11—C16—H16B	109.7	C36—C31—C32	118.79 (13)
C15—C16—H16B	109.7	C36—C31—C37	121.27 (12)
H16A—C16—H16B	108.2	C32—C31—C37	119.93 (13)
C146—C141—C142	117.13 (12)	C33—C32—C31	120.19 (14)
C146—C141—N14	120.41 (11)	C33—C32—H32	119.9
C142—C141—N14	122.38 (12)	C31—C32—H32	119.9
C143—C142—C141	120.97 (13)	C32—C33—C34	121.44 (15)
C143—C142—H142	119.5	C32—C33—H33	119.3
C141—C142—H142	119.5	C34—C33—H33	119.3
C142—C143—C144	121.15 (14)	C33—C34—C35	118.01 (14)
C142—C143—H143	119.4	C33—C34—C38	121.66 (16)
C144—C143—H143	119.4	C35—C34—C38	120.33 (16)
O144—C144—C145	125.25 (14)	C36—C35—C34	121.09 (14)
O144—C144—C143	115.95 (13)	C36—C35—H35	119.5
C145—C144—C143	118.79 (13)	C34—C35—H35	119.5
C144—C145—C146	120.21 (13)	C31—C36—C35	120.44 (14)
C144—C145—H145	119.9	C31—C36—H36	119.8
C146—C145—H145	119.9	C35—C36—H36	119.8
C145—C146—C141	121.74 (13)	O32—C37—O31	124.57 (13)
C145—C146—H146	119.1	O32—C37—C31	117.82 (13)
C141—C146—H146	119.1	O31—C37—C31	117.61 (12)
C144—O144—C147	117.49 (13)	C34—C38—H38A	109.5
O144—C147—H17A	109.5	C34—C38—H38B	109.5
O144—C147—H17B	109.5	H38A—C38—H38B	109.5
H17A—C147—H17B	109.5	C34—C38—H38C	109.5
O144—C147—H17C	109.5	H38A—C38—H38C	109.5
H17A—C147—H17C	109.5	H38B—C38—H38C	109.5
H17B—C147—H17C	109.5	C46—C41—C42	118.81 (12)
C22—N21—C26	109.64 (10)	C46—C41—C47	121.00 (12)
C22—N21—H21	110.7 (9)	C42—C41—C47	120.18 (12)
C26—N21—H21	111.8 (9)	C43—C42—C41	120.21 (14)
C22—N21—H22	106.9 (10)	C43—C42—H42	119.9
C26—N21—H22	110.2 (10)	C41—C42—H42	119.9
H21—N21—H22	107.5 (13)	C44—C43—C42	121.24 (15)
N21—C22—C23	109.97 (11)	C44—C43—H43	119.4
N21—C22—H22A	109.7	C42—C43—H43	119.4
C23—C22—H22A	109.7	C43—C44—C45	118.30 (14)
N21—C22—H22B	109.7	C43—C44—C48	121.61 (16)
C23—C22—H22B	109.7	C45—C44—C48	120.08 (16)
H22A—C22—H22B	108.2	C44—C45—C46	121.03 (14)
N24—C23—C22	112.03 (11)	C44—C45—H45	119.5
N24—C23—H23A	109.2	C46—C45—H45	119.5
C22—C23—H23A	109.2	C45—C46—C41	120.40 (13)
N24—C23—H23B	109.2	C45—C46—H46	119.8
C22—C23—H23B	109.2	C41—C46—H46	119.8
H23A—C23—H23B	107.9	O42—C47—O41	123.97 (13)
C241—N24—C25	114.88 (10)	O42—C47—C41	117.63 (14)
C241—N24—C23	113.35 (10)	O41—C47—C41	118.40 (12)
C25—N24—C23	110.62 (10)	C44—C48—H48A	109.5
N24—C25—C26	111.20 (11)	C44—C48—H48B	109.5
N24—C25—H25A	109.4	H48A—C48—H48B	109.5
C26—C25—H25A	109.4	C44—C48—H48C	109.5
N24—C25—H25B	109.4	H48A—C48—H48C	109.5
C26—C25—H25B	109.4	H48B—C48—H48C	109.5
H25A—C25—H25B	108.0	H51—O51—H52	105.5 (16)
N21—C26—C25	110.03 (10)	H61—O61—H62	106.6 (16)
			
C16—N11—C12—C13	−57.49 (14)	C241—C242—C243—C244	−0.2 (3)
N11—C12—C13—N14	56.22 (15)	C242—C243—C244—C245	1.7 (3)
C12—C13—N14—C141	171.77 (11)	C242—C243—C244—O244	−177.89 (16)
C12—C13—N14—C15	−55.02 (15)	C243—C244—C245—C246	−1.6 (3)
C141—N14—C15—C16	−171.14 (10)	O244—C244—C245—C246	177.91 (16)
C13—N14—C15—C16	55.58 (14)	C242—C241—C246—C245	1.3 (2)
C12—N11—C16—C15	58.26 (14)	N24—C241—C246—C245	179.21 (14)
N14—C15—C16—N11	−57.35 (14)	C244—C245—C246—C241	0.1 (3)
C13—N14—C141—C146	177.99 (12)	C245—C244—O244—C247	0.4 (3)
C15—N14—C141—C146	46.58 (16)	C243—C244—O244—C247	179.97 (18)
C13—N14—C141—C142	−5.45 (18)	C36—C31—C32—C33	1.5 (2)
C15—N14—C141—C142	−136.85 (14)	C37—C31—C32—C33	−177.47 (13)
C146—C141—C142—C143	1.3 (2)	C31—C32—C33—C34	−0.6 (2)
N14—C141—C142—C143	−175.37 (14)	C32—C33—C34—C35	−1.2 (2)
C141—C142—C143—C144	−1.2 (3)	C32—C33—C34—C38	178.95 (15)
C142—C143—C144—O144	−179.29 (15)	C33—C34—C35—C36	2.1 (2)
C142—C143—C144—C145	0.2 (3)	C38—C34—C35—C36	−178.08 (15)
O144—C144—C145—C146	−179.99 (15)	C32—C31—C36—C35	−0.7 (2)
C143—C144—C145—C146	0.6 (2)	C37—C31—C36—C35	178.31 (12)
C144—C145—C146—C141	−0.4 (2)	C34—C35—C36—C31	−1.1 (2)
C142—C141—C146—C145	−0.5 (2)	C36—C31—C37—O32	168.27 (13)
N14—C141—C146—C145	176.20 (13)	C32—C31—C37—O32	−12.75 (19)
C145—C144—O144—C147	−4.8 (3)	C36—C31—C37—O31	−12.27 (18)
C143—C144—O144—C147	174.67 (17)	C32—C31—C37—O31	166.71 (13)
C26—N21—C22—C23	−57.46 (14)	C46—C41—C42—C43	0.7 (2)
N21—C22—C23—N24	56.44 (15)	C47—C41—C42—C43	179.74 (13)
C22—C23—N24—C241	173.71 (11)	C41—C42—C43—C44	−0.5 (2)
C22—C23—N24—C25	−55.59 (14)	C42—C43—C44—C45	0.2 (2)
C241—N24—C25—C26	−173.81 (10)	C42—C43—C44—C48	179.55 (15)
C23—N24—C25—C26	56.30 (13)	C43—C44—C45—C46	0.0 (2)
C22—N21—C26—C25	58.68 (14)	C48—C44—C45—C46	−179.38 (15)
N24—C25—C26—N21	−58.38 (14)	C44—C45—C46—C41	0.2 (2)
C25—N24—C241—C246	6.68 (18)	C42—C41—C46—C45	−0.5 (2)
C23—N24—C241—C246	135.22 (14)	C47—C41—C46—C45	−179.56 (12)
C25—N24—C241—C242	−175.46 (13)	C46—C41—C47—O42	175.08 (13)
C23—N24—C241—C242	−46.92 (17)	C42—C41—C47—O42	−3.96 (19)
C246—C241—C242—C243	−1.2 (2)	C46—C41—C47—O41	−4.69 (19)
N24—C241—C242—C243	−179.21 (15)	C42—C41—C47—O41	176.28 (12)

### 4-(4-Methoxyphenyl)piperazin-1-ium 4-methylbenzoate monohydrate (I) Hydrogen-bond geometry (Å, º)

*Cg*1 and *Cg*2 represent the centroids of the C31–C36 and C41–C46 rings, respectively.

**Table:**

*D*—H···*A*	*D*—H	H···*A*	*D*···*A*	*D*—H···*A*
N11—H11···O31	0.946 (16)	1.793 (16)	2.7366 (16)	174.5 (13)
N11—H12···O61^i^	0.917 (15)	1.891 (15)	2.7969 (16)	169.2 (13)
N21—H21···O41	0.949 (16)	1.813 (16)	2.7592 (16)	174.1 (14)
N21—H22···O51^i^	0.904 (15)	1.909 (15)	2.8047 (16)	170.9 (13)
O51—H51···O41^ii^	0.89 (2)	1.89 (2)	2.7712 (16)	170.0 (18)
O51—H52···O32	0.918 (18)	1.739 (18)	2.6539 (16)	174.3 (15)
O61—H61···O42	0.906 (19)	1.733 (19)	2.6315 (16)	171.2 (15)
O61—H62···O31	0.92 (2)	1.85 (2)	2.7593 (15)	169.1 (19)
C22—H22*B*···O41^iii^	0.99	2.56	3.5266 (19)	167
C142—H142···*Cg*1^i^	0.95	2.83	3.5976 (16)	138
C146—H146···*Cg*2^iv^	0.95	2.73	3.5478 (16)	145

Symmetry codes: (i) −*x*+2, −*y*+1, −*z*+1; (ii) *x*, *y*+1, *z*; (iii) −*x*+1, −*y*, −*z*+1; (iv) −*x*+1, −*y*+1, −*z*+1.

### Bis[4-(4-methoxyphenyl)piperazin-1-ium] benzene-1,2-dicarboxylate (II) Crystal data

**Table:**

2C_11_H_17_N_2_O^+^·C_8_H_4_O_4_^2^^−^	*D*_x_ = 1.332 Mg m^−^^3^
*M**_r_* = 550.64	Cu *K*α radiation, λ = 1.54178 Å
Orthorhombic, *P**n**a*2_1_	Cell parameters from 10053 reflections
*a* = 17.8424 (4) Å	θ = 5.0–70.1°
*b* = 8.8124 (2) Å	µ = 0.76 mm^−^^1^
*c* = 34.9337 (9) Å	*T* = 180 K
*V* = 5492.8 (2) Å^3^	Plate, colourless
*Z* = 8	0.26 × 0.24 × 0.04 mm
*F*(000) = 2352	

### Bis[4-(4-methoxyphenyl)piperazin-1-ium] benzene-1,2-dicarboxylate (II) Data collection

**Table:**

Bruker D8 Venture dual source diffractometer	10053 independent reflections
Radiation source: microsource	9727 reflections with *I* > 2σ(*I*)
Multilayer optics monochromator	*R*_int_ = 0.031
φ and ω scans	θ_max_ = 70.1°, θ_min_ = 5.0°
Absorption correction: multi-scan (SADABS; Krause *et al.*, 2015)	*h* = −21→21
*T*_min_ = 0.814, *T*_max_ = 0.942	*k* = −10→10
36488 measured reflections	*l* = −41→42

### Bis[4-(4-methoxyphenyl)piperazin-1-ium] benzene-1,2-dicarboxylate (II) Refinement

**Table:**

Refinement on *F*^2^	H atoms treated by a mixture of independent and constrained refinement
Least-squares matrix: full	*w* = 1/[σ^2^(*F*_o_^2^) + (0.0511*P*)^2^ + 1.5447*P*] where *P* = (*F*_o_^2^ + 2*F*_c_^2^)/3
*R*[*F*^2^ > 2σ(*F*^2^)] = 0.037	(Δ/σ)_max_ = 0.001
*wR*(*F*^2^) = 0.098	Δρ_max_ = 0.27 e Å^−^^3^
*S* = 1.07	Δρ_min_ = −0.17 e Å^−^^3^
10053 reflections	Extinction correction: SHELXL, Fc^*^=kFc[1+0.001xFc^2^λ^3^/sin(2θ)]^-1/4^
750 parameters	Extinction coefficient: 0.0018 (2)
1 restraint	Absolute structure: Flack *x* determined using 4511 quotients [(*I*^+^)-(*I*^-^)]/[(*I*^+^)+(*I*^-^)] (Parsons *et al.*, 2013)
Primary atom site location: dual	Absolute structure parameter: 0.45 (5)
Hydrogen site location: mixed	

### Bis[4-(4-methoxyphenyl)piperazin-1-ium] benzene-1,2-dicarboxylate (II) Special details

**Table:**

Geometry. All esds (except the esd in the dihedral angle between two l.s. planes) are estimated using the full covariance matrix. The cell esds are taken into account individually in the estimation of esds in distances, angles and torsion angles; correlations between esds in cell parameters are only used when they are defined by crystal symmetry. An approximate (isotropic) treatment of cell esds is used for estimating esds involving l.s. planes.

### Bis[4-(4-methoxyphenyl)piperazin-1-ium] benzene-1,2-dicarboxylate (II) Fractional atomic coordinates and isotropic or equivalent isotropic displacement parameters (Å^2^)

**Table:**

	*x*	*y*	*z*	*U*_iso_*/*U*_eq_	
N11	0.87134 (14)	0.7550 (3)	0.46161 (8)	0.0272 (5)	
H11	0.8642 (18)	0.778 (4)	0.4891 (11)	0.033*	
H12	0.909 (2)	0.799 (4)	0.4563 (10)	0.033*	
C12	0.80552 (16)	0.8091 (3)	0.43903 (9)	0.0320 (6)	
H12A	0.8019	0.9209	0.4409	0.038*	
H12B	0.7590	0.7650	0.4498	0.038*	
C13	0.81286 (17)	0.7636 (3)	0.39740 (8)	0.0285 (6)	
H13A	0.7669	0.7931	0.3834	0.034*	
H13B	0.8558	0.8178	0.3857	0.034*	
N14	0.82434 (12)	0.5999 (2)	0.39400 (6)	0.0235 (5)	
C15	0.89338 (15)	0.5558 (3)	0.41323 (8)	0.0267 (6)	
H15A	0.9362	0.6126	0.4023	0.032*	
H15B	0.9026	0.4460	0.4095	0.032*	
C16	0.88572 (16)	0.5909 (3)	0.45548 (8)	0.0297 (6)	
H16A	0.8439	0.5310	0.4664	0.036*	
H16B	0.9323	0.5612	0.4689	0.036*	
C141	0.81224 (15)	0.5400 (3)	0.35683 (7)	0.0230 (5)	
C142	0.73982 (16)	0.5497 (4)	0.34152 (9)	0.0328 (6)	
H142	0.7014	0.5990	0.3557	0.039*	
C143	0.72360 (18)	0.4888 (4)	0.30617 (10)	0.0362 (7)	
H143	0.6742	0.4973	0.2962	0.043*	
C144	0.77848 (17)	0.4148 (3)	0.28482 (8)	0.0304 (6)	
C145	0.84993 (16)	0.4047 (3)	0.29947 (8)	0.0298 (6)	
H145	0.8880	0.3546	0.2853	0.036*	
C146	0.86657 (15)	0.4677 (3)	0.33516 (8)	0.0269 (6)	
H146	0.9163	0.4609	0.3448	0.032*	
O144	0.75569 (14)	0.3577 (3)	0.25005 (7)	0.0448 (6)	
C147	0.80702 (19)	0.2601 (4)	0.23098 (9)	0.0379 (7)	
H17A	0.8533	0.3157	0.2254	0.057*	
H17B	0.7847	0.2242	0.2070	0.057*	
H17C	0.8185	0.1730	0.2474	0.057*	
N21	0.62871 (13)	0.7455 (3)	1.03620 (8)	0.0285 (5)	
H21	0.6222 (19)	0.722 (4)	1.0088 (11)	0.034*	
H22	0.676 (2)	0.689 (4)	1.0436 (10)	0.034*	
C22	0.56305 (16)	0.6921 (3)	1.05899 (9)	0.0326 (6)	
H22A	0.5589	0.5804	1.0569	0.039*	
H22B	0.5166	0.7372	1.0485	0.039*	
C23	0.57104 (16)	0.7362 (3)	1.10062 (8)	0.0286 (6)	
H23A	0.5250	0.7077	1.1147	0.034*	
H23B	0.6136	0.6803	1.1121	0.034*	
N24	0.58379 (12)	0.8995 (2)	1.10434 (6)	0.0237 (5)	
C25	0.65261 (16)	0.9418 (3)	1.08482 (8)	0.0274 (6)	
H25A	0.6952	0.8828	1.0953	0.033*	
H25B	0.6630	1.0510	1.0888	0.033*	
C26	0.64373 (17)	0.9093 (3)	1.04258 (8)	0.0310 (6)	
H26A	0.6018	0.9701	1.0322	0.037*	
H26B	0.6900	0.9390	1.0289	0.037*	
C241	0.57153 (15)	0.9597 (3)	1.14149 (8)	0.0230 (5)	
C242	0.49942 (16)	0.9532 (3)	1.15652 (9)	0.0304 (6)	
H242	0.4610	0.9041	1.1423	0.036*	
C243	0.48233 (17)	1.0164 (4)	1.19163 (10)	0.0356 (7)	
H243	0.4327	1.0100	1.2013	0.043*	
C244	0.53764 (17)	1.0895 (3)	1.21301 (8)	0.0295 (6)	
C245	0.60958 (16)	1.0977 (3)	1.19861 (8)	0.0289 (6)	
H245	0.6477	1.1476	1.2128	0.035*	
C246	0.62640 (15)	1.0326 (3)	1.16312 (8)	0.0273 (6)	
H246	0.6762	1.0382	1.1536	0.033*	
O244	0.51493 (13)	1.1490 (3)	1.24745 (6)	0.0407 (5)	
C247	0.5690 (2)	1.2308 (4)	1.26905 (10)	0.0433 (8)	
H27A	0.6094	1.1621	1.2769	0.065*	
H27B	0.5451	1.2741	1.2918	0.065*	
H27C	0.5898	1.3127	1.2533	0.065*	
N31	0.59650 (13)	0.7174 (3)	0.52626 (7)	0.0261 (5)	
H31	0.5784 (19)	0.718 (4)	0.5010 (10)	0.031*	
H32	0.640 (2)	0.774 (4)	0.5272 (10)	0.031*	
C32	0.61659 (16)	0.5555 (3)	0.53320 (8)	0.0291 (6)	
H32A	0.6589	0.5267	0.5164	0.035*	
H32B	0.5733	0.4901	0.5267	0.035*	
C33	0.63847 (16)	0.5297 (3)	0.57459 (8)	0.0286 (6)	
H33A	0.6491	0.4207	0.5788	0.034*	
H33B	0.6845	0.5878	0.5805	0.034*	
N34	0.57770 (13)	0.5783 (3)	0.59990 (6)	0.0247 (5)	
C35	0.56205 (17)	0.7403 (3)	0.59425 (9)	0.0284 (6)	
H35A	0.6076	0.8001	0.6001	0.034*	
H35B	0.5220	0.7727	0.6121	0.034*	
C36	0.53783 (16)	0.7706 (3)	0.55351 (8)	0.0291 (6)	
H36A	0.4901	0.7172	0.5483	0.035*	
H36B	0.5293	0.8808	0.5499	0.035*	
C341	0.58380 (15)	0.5329 (3)	0.63868 (8)	0.0245 (5)	
C342	0.64566 (17)	0.4557 (3)	0.65329 (8)	0.0312 (6)	
H342	0.6874	0.4372	0.6371	0.037*	
C343	0.64786 (19)	0.4050 (4)	0.69096 (9)	0.0377 (7)	
H343	0.6901	0.3504	0.7001	0.045*	
C344	0.58839 (19)	0.4345 (3)	0.71498 (8)	0.0342 (7)	
C345	0.52757 (19)	0.5138 (4)	0.70097 (10)	0.0395 (7)	
H345	0.4868	0.5360	0.7176	0.047*	
C346	0.52465 (17)	0.5612 (4)	0.66353 (9)	0.0351 (7)	
H346	0.4817	0.6140	0.6545	0.042*	
O344	0.58749 (16)	0.3975 (3)	0.75337 (6)	0.0499 (6)	
C347	0.6144 (2)	0.2511 (4)	0.76343 (10)	0.0434 (8)	
H37A	0.5844	0.1736	0.7504	0.065*	
H37B	0.6103	0.2372	0.7912	0.065*	
H37C	0.6670	0.2416	0.7557	0.065*	
N41	0.35347 (13)	0.7834 (3)	0.96744 (7)	0.0284 (5)	
H41	0.3333 (19)	0.780 (4)	0.9940 (11)	0.034*	
H42	0.394 (2)	0.723 (4)	0.9660 (10)	0.034*	
C42	0.37614 (16)	0.9436 (3)	0.96097 (9)	0.0311 (6)	
H42A	0.4193	0.9683	0.9777	0.037*	
H42B	0.3342	1.0115	0.9680	0.037*	
C43	0.39750 (16)	0.9709 (3)	0.91972 (9)	0.0309 (6)	
H43A	0.4100	1.0794	0.9160	0.037*	
H43B	0.4424	0.9101	0.9132	0.037*	
N44	0.33606 (13)	0.9291 (2)	0.89465 (7)	0.0267 (5)	
C45	0.31864 (16)	0.7675 (3)	0.89905 (9)	0.0306 (6)	
H45A	0.3634	0.7062	0.8926	0.037*	
H45B	0.2779	0.7391	0.8812	0.037*	
C46	0.29473 (16)	0.7340 (3)	0.93967 (10)	0.0315 (6)	
H46A	0.2472	0.7877	0.9453	0.038*	
H46B	0.2857	0.6237	0.9426	0.038*	
C441	0.33856 (16)	0.9847 (3)	0.85647 (8)	0.0266 (6)	
C442	0.39903 (16)	1.0619 (3)	0.84117 (8)	0.0294 (6)	
H442	0.4442	1.0682	0.8555	0.035*	
C443	0.39546 (17)	1.1310 (3)	0.80501 (9)	0.0321 (6)	
H443	0.4375	1.1848	0.7953	0.038*	
C444	0.33068 (17)	1.1208 (3)	0.78350 (8)	0.0322 (6)	
C445	0.27056 (18)	1.0369 (4)	0.79782 (9)	0.0351 (7)	
H445	0.2265	1.0252	0.7828	0.042*	
C446	0.27444 (16)	0.9710 (3)	0.83348 (9)	0.0315 (6)	
H446	0.2327	0.9150	0.8427	0.038*	
O447	0.31901 (13)	1.1870 (2)	0.74835 (6)	0.0407 (5)	
C447	0.3794 (2)	1.2730 (4)	0.73265 (10)	0.0438 (8)	
H47A	0.4236	1.2078	0.7299	0.066*	
H47B	0.3912	1.3579	0.7497	0.066*	
H47C	0.3649	1.3123	0.7075	0.066*	
C51	0.77813 (14)	0.9274 (3)	0.58429 (7)	0.0218 (5)	
C52	0.72145 (14)	1.0348 (3)	0.59017 (8)	0.0230 (5)	
C53	0.70626 (16)	1.0837 (3)	0.62739 (8)	0.0290 (6)	
H53	0.6675	1.1555	0.6316	0.035*	
C54	0.74701 (18)	1.0288 (3)	0.65831 (9)	0.0343 (6)	
H54	0.7356	1.0620	0.6835	0.041*	
C55	0.80426 (18)	0.9257 (3)	0.65224 (8)	0.0335 (6)	
H55	0.8331	0.8893	0.6732	0.040*	
C56	0.81934 (15)	0.8757 (3)	0.61533 (8)	0.0269 (6)	
H56	0.8586	0.8047	0.6113	0.032*	
C57	0.79259 (14)	0.8587 (3)	0.54501 (7)	0.0216 (5)	
O51	0.85883 (12)	0.8253 (3)	0.53699 (6)	0.0442 (6)	
O52	0.73774 (11)	0.8367 (3)	0.52362 (6)	0.0339 (5)	
C58	0.67939 (15)	1.1118 (3)	0.55757 (8)	0.0258 (6)	
O53	0.72016 (11)	1.1881 (2)	0.53494 (6)	0.0315 (4)	
O54	0.61057 (11)	1.1038 (3)	0.55663 (7)	0.0431 (6)	
C61	0.53754 (14)	0.5763 (3)	0.91267 (7)	0.0203 (5)	
C62	0.48063 (14)	0.4705 (3)	0.90566 (7)	0.0210 (5)	
C63	0.46708 (15)	0.4225 (3)	0.86845 (8)	0.0261 (6)	
H63	0.4280	0.3518	0.8637	0.031*	
C64	0.50944 (16)	0.4758 (3)	0.83823 (8)	0.0289 (6)	
H64	0.4992	0.4423	0.8129	0.035*	
C65	0.56708 (16)	0.5782 (3)	0.84487 (8)	0.0290 (6)	
H65	0.5968	0.6142	0.8242	0.035*	
C66	0.58113 (15)	0.6279 (3)	0.88205 (8)	0.0245 (5)	
H66	0.6208	0.6976	0.8866	0.029*	
C67	0.55033 (15)	0.6439 (3)	0.95187 (7)	0.0234 (5)	
O61	0.61608 (11)	0.6799 (3)	0.96078 (6)	0.0408 (6)	
O62	0.49458 (10)	0.6629 (2)	0.97323 (6)	0.0325 (5)	
C68	0.43777 (14)	0.3927 (3)	0.93738 (8)	0.0237 (5)	
O63	0.47775 (10)	0.3110 (2)	0.95979 (6)	0.0296 (4)	
O64	0.36920 (11)	0.4038 (3)	0.93907 (7)	0.0391 (5)	

### Bis[4-(4-methoxyphenyl)piperazin-1-ium] benzene-1,2-dicarboxylate (II) Atomic displacement parameters (Å^2^)

**Table:**

	*U*^11^	*U*^22^	*U*^33^	*U*^12^	*U*^13^	*U*^23^
N11	0.0225 (12)	0.0352 (14)	0.0239 (13)	−0.0028 (9)	0.0018 (9)	−0.0044 (10)
C12	0.0274 (13)	0.0363 (16)	0.0322 (15)	0.0080 (12)	−0.0026 (12)	−0.0077 (12)
C13	0.0339 (15)	0.0227 (13)	0.0289 (16)	0.0054 (11)	−0.0026 (11)	−0.0008 (11)
N14	0.0225 (11)	0.0220 (11)	0.0260 (11)	0.0025 (8)	−0.0030 (9)	0.0003 (9)
C15	0.0245 (13)	0.0246 (13)	0.0310 (15)	0.0054 (11)	−0.0033 (11)	0.0005 (11)
C16	0.0307 (14)	0.0315 (15)	0.0270 (15)	−0.0028 (12)	−0.0048 (11)	0.0054 (11)
C141	0.0237 (13)	0.0200 (12)	0.0254 (13)	−0.0003 (10)	−0.0019 (10)	0.0027 (10)
C142	0.0214 (13)	0.0361 (15)	0.0408 (17)	0.0055 (11)	−0.0035 (12)	−0.0078 (13)
C143	0.0283 (15)	0.0392 (17)	0.0410 (18)	0.0071 (13)	−0.0119 (13)	−0.0059 (14)
C144	0.0372 (15)	0.0284 (14)	0.0257 (14)	0.0041 (12)	−0.0049 (11)	0.0011 (11)
C145	0.0313 (15)	0.0303 (14)	0.0279 (14)	0.0047 (12)	0.0035 (11)	0.0003 (11)
C146	0.0227 (13)	0.0294 (14)	0.0286 (14)	0.0008 (10)	−0.0016 (10)	0.0011 (11)
O144	0.0509 (14)	0.0496 (13)	0.0339 (12)	0.0120 (11)	−0.0116 (10)	−0.0124 (10)
C147	0.0454 (18)	0.0378 (17)	0.0304 (17)	−0.0048 (14)	0.0010 (13)	−0.0046 (13)
N21	0.0239 (12)	0.0360 (14)	0.0255 (13)	0.0028 (10)	−0.0014 (9)	−0.0068 (10)
C22	0.0287 (14)	0.0328 (16)	0.0363 (16)	−0.0043 (12)	0.0029 (12)	−0.0096 (12)
C23	0.0308 (14)	0.0245 (14)	0.0303 (16)	−0.0054 (11)	0.0044 (11)	−0.0027 (11)
N24	0.0243 (11)	0.0232 (11)	0.0237 (11)	−0.0015 (9)	0.0022 (8)	−0.0015 (8)
C25	0.0307 (14)	0.0270 (13)	0.0245 (14)	−0.0036 (11)	0.0073 (11)	−0.0010 (10)
C26	0.0324 (15)	0.0352 (16)	0.0254 (14)	0.0030 (12)	0.0029 (11)	0.0037 (12)
C241	0.0246 (12)	0.0215 (12)	0.0228 (13)	0.0015 (10)	0.0005 (10)	0.0023 (10)
C242	0.0252 (13)	0.0311 (14)	0.0349 (16)	−0.0031 (11)	−0.0001 (11)	−0.0046 (12)
C243	0.0264 (15)	0.0391 (16)	0.0415 (18)	−0.0004 (13)	0.0109 (12)	−0.0053 (13)
C244	0.0362 (15)	0.0254 (14)	0.0268 (14)	0.0045 (12)	0.0080 (11)	−0.0002 (11)
C245	0.0323 (15)	0.0278 (14)	0.0265 (14)	−0.0031 (11)	−0.0011 (11)	−0.0015 (11)
C246	0.0249 (13)	0.0285 (14)	0.0286 (14)	−0.0027 (11)	0.0036 (10)	0.0018 (11)
O244	0.0462 (13)	0.0436 (12)	0.0324 (11)	0.0027 (10)	0.0121 (10)	−0.0096 (10)
C247	0.064 (2)	0.0368 (17)	0.0291 (17)	0.0086 (16)	−0.0008 (15)	−0.0085 (13)
N31	0.0213 (11)	0.0293 (12)	0.0277 (12)	−0.0055 (10)	−0.0030 (9)	0.0005 (9)
C32	0.0314 (14)	0.0299 (14)	0.0261 (14)	0.0022 (11)	0.0014 (11)	−0.0025 (11)
C33	0.0277 (14)	0.0298 (14)	0.0283 (14)	0.0071 (11)	0.0021 (11)	−0.0036 (11)
N34	0.0249 (11)	0.0250 (11)	0.0243 (11)	0.0035 (9)	−0.0006 (9)	−0.0028 (9)
C35	0.0308 (14)	0.0226 (13)	0.0317 (15)	0.0038 (11)	0.0021 (11)	−0.0029 (11)
C36	0.0255 (14)	0.0298 (14)	0.0321 (16)	0.0002 (11)	−0.0011 (11)	0.0015 (11)
C341	0.0263 (13)	0.0219 (12)	0.0252 (13)	−0.0009 (10)	−0.0012 (10)	−0.0036 (10)
C342	0.0304 (14)	0.0353 (15)	0.0280 (15)	0.0096 (12)	0.0045 (11)	−0.0021 (12)
C343	0.0406 (17)	0.0391 (17)	0.0334 (16)	0.0147 (13)	−0.0032 (13)	−0.0021 (13)
C344	0.0477 (18)	0.0302 (14)	0.0246 (14)	0.0027 (13)	0.0026 (12)	−0.0023 (11)
C345	0.0422 (17)	0.0413 (17)	0.0352 (17)	0.0090 (14)	0.0097 (13)	−0.0011 (13)
C346	0.0304 (15)	0.0411 (16)	0.0337 (16)	0.0101 (13)	0.0030 (12)	0.0018 (13)
O344	0.0782 (18)	0.0450 (12)	0.0265 (11)	0.0175 (12)	0.0065 (11)	0.0069 (10)
C347	0.063 (2)	0.0353 (17)	0.0322 (18)	−0.0003 (15)	−0.0069 (15)	0.0080 (13)
N41	0.0229 (11)	0.0298 (12)	0.0323 (13)	0.0036 (10)	0.0035 (9)	0.0000 (10)
C42	0.0279 (14)	0.0310 (15)	0.0342 (15)	−0.0008 (11)	−0.0022 (11)	−0.0042 (12)
C43	0.0274 (14)	0.0315 (15)	0.0337 (15)	−0.0063 (12)	−0.0032 (11)	−0.0015 (12)
N44	0.0249 (11)	0.0239 (11)	0.0313 (12)	−0.0033 (9)	−0.0018 (9)	−0.0027 (9)
C45	0.0286 (14)	0.0253 (14)	0.0379 (17)	−0.0005 (11)	−0.0013 (12)	−0.0059 (12)
C46	0.0233 (13)	0.0267 (14)	0.0445 (18)	−0.0036 (11)	0.0023 (12)	−0.0016 (12)
C441	0.0272 (14)	0.0242 (13)	0.0282 (14)	0.0005 (11)	0.0009 (10)	−0.0065 (11)
C442	0.0247 (13)	0.0286 (14)	0.0350 (16)	−0.0020 (11)	−0.0027 (11)	−0.0044 (11)
C443	0.0319 (15)	0.0301 (15)	0.0342 (15)	−0.0029 (12)	0.0024 (12)	−0.0029 (12)
C444	0.0390 (16)	0.0291 (14)	0.0287 (14)	0.0013 (12)	−0.0013 (12)	−0.0057 (11)
C445	0.0330 (16)	0.0376 (17)	0.0346 (16)	0.0006 (13)	−0.0079 (12)	−0.0075 (12)
C446	0.0257 (14)	0.0353 (15)	0.0337 (15)	−0.0051 (11)	−0.0004 (11)	−0.0041 (12)
O447	0.0459 (12)	0.0435 (12)	0.0328 (11)	0.0014 (10)	−0.0040 (9)	0.0028 (9)
C447	0.058 (2)	0.0386 (17)	0.0344 (18)	0.0009 (16)	0.0035 (15)	0.0054 (14)
C51	0.0206 (12)	0.0217 (12)	0.0230 (13)	−0.0033 (10)	−0.0008 (10)	−0.0014 (10)
C52	0.0206 (12)	0.0213 (12)	0.0271 (13)	−0.0047 (9)	0.0028 (10)	−0.0021 (10)
C53	0.0256 (14)	0.0299 (14)	0.0314 (15)	−0.0030 (11)	0.0065 (11)	−0.0042 (11)
C54	0.0424 (17)	0.0340 (16)	0.0266 (14)	−0.0041 (13)	0.0063 (12)	−0.0063 (12)
C55	0.0444 (17)	0.0321 (14)	0.0239 (14)	−0.0039 (12)	−0.0060 (12)	0.0014 (11)
C56	0.0273 (13)	0.0232 (13)	0.0303 (15)	0.0014 (11)	−0.0001 (11)	−0.0009 (10)
C57	0.0206 (12)	0.0208 (12)	0.0233 (13)	−0.0002 (9)	0.0015 (10)	−0.0021 (10)
O51	0.0259 (10)	0.0750 (17)	0.0317 (11)	0.0149 (10)	−0.0033 (9)	−0.0179 (11)
O52	0.0216 (10)	0.0508 (13)	0.0292 (10)	−0.0074 (9)	0.0000 (8)	−0.0124 (9)
C58	0.0206 (13)	0.0235 (13)	0.0334 (14)	0.0003 (10)	−0.0014 (10)	−0.0036 (11)
O53	0.0265 (10)	0.0337 (11)	0.0344 (11)	−0.0034 (8)	−0.0065 (8)	0.0084 (9)
O54	0.0178 (10)	0.0556 (14)	0.0558 (14)	0.0019 (9)	−0.0016 (9)	0.0104 (12)
C61	0.0178 (11)	0.0188 (12)	0.0244 (13)	0.0037 (9)	−0.0009 (9)	−0.0011 (10)
C62	0.0168 (11)	0.0222 (12)	0.0240 (13)	0.0031 (9)	0.0000 (9)	−0.0011 (10)
C63	0.0250 (13)	0.0247 (13)	0.0286 (14)	0.0007 (10)	−0.0049 (10)	−0.0032 (11)
C64	0.0348 (15)	0.0321 (14)	0.0198 (13)	0.0065 (12)	−0.0033 (11)	−0.0026 (11)
C65	0.0317 (14)	0.0302 (14)	0.0252 (14)	0.0025 (11)	0.0043 (11)	0.0054 (11)
C66	0.0256 (13)	0.0211 (12)	0.0268 (13)	0.0008 (10)	0.0031 (10)	0.0006 (10)
C67	0.0223 (13)	0.0224 (12)	0.0254 (13)	0.0014 (10)	−0.0005 (10)	−0.0015 (10)
O61	0.0218 (10)	0.0699 (16)	0.0307 (11)	−0.0123 (10)	0.0018 (8)	−0.0169 (11)
O62	0.0207 (9)	0.0486 (12)	0.0281 (10)	0.0055 (8)	0.0001 (8)	−0.0112 (9)
C68	0.0208 (12)	0.0238 (13)	0.0266 (13)	−0.0007 (10)	0.0008 (10)	−0.0028 (10)
O63	0.0248 (9)	0.0344 (11)	0.0296 (10)	0.0059 (8)	0.0050 (8)	0.0063 (8)
O64	0.0181 (9)	0.0556 (14)	0.0436 (12)	−0.0006 (9)	0.0027 (9)	0.0092 (10)

### Bis[4-(4-methoxyphenyl)piperazin-1-ium] benzene-1,2-dicarboxylate (II) Geometric parameters (Å, º)

**Table:**

N11—C16	1.484 (4)	C36—H36B	0.9900
N11—C12	1.493 (4)	C341—C346	1.389 (4)
N11—H11	0.99 (4)	C341—C342	1.394 (4)
N11—H12	0.79 (4)	C342—C343	1.390 (4)
C12—C13	1.514 (4)	C342—H342	0.9500
C12—H12A	0.9900	C343—C344	1.378 (4)
C12—H12B	0.9900	C343—H343	0.9500
C13—N14	1.462 (3)	C344—O344	1.380 (4)
C13—H13A	0.9900	C344—C345	1.380 (5)
C13—H13B	0.9900	C345—C346	1.374 (5)
N14—C141	1.418 (3)	C345—H345	0.9500
N14—C15	1.456 (3)	C346—H346	0.9500
C15—C16	1.514 (4)	O344—C347	1.420 (4)
C15—H15A	0.9900	C347—H37A	0.9800
C15—H15B	0.9900	C347—H37B	0.9800
C16—H16A	0.9900	C347—H37C	0.9800
C16—H16B	0.9900	N41—C42	1.485 (4)
C141—C146	1.385 (4)	N41—C46	1.493 (4)
C141—C142	1.401 (4)	N41—H41	1.00 (4)
C142—C143	1.378 (5)	N41—H42	0.91 (4)
C142—H142	0.9500	C42—C43	1.510 (4)
C143—C144	1.393 (4)	C42—H42A	0.9900
C143—H143	0.9500	C42—H42B	0.9900
C144—O144	1.376 (4)	C43—N44	1.451 (4)
C144—C145	1.377 (4)	C43—H43A	0.9900
C145—C146	1.397 (4)	C43—H43B	0.9900
C145—H145	0.9500	N44—C441	1.422 (4)
C146—H146	0.9500	N44—C45	1.465 (4)
O144—C147	1.422 (4)	C45—C46	1.511 (5)
C147—H17A	0.9800	C45—H45A	0.9900
C147—H17B	0.9800	C45—H45B	0.9900
C147—H17C	0.9800	C46—H46A	0.9900
N21—C26	1.485 (4)	C46—H46B	0.9900
N21—C22	1.493 (4)	C441—C442	1.383 (4)
N21—H21	0.99 (4)	C441—C446	1.403 (4)
N21—H22	1.01 (4)	C442—C443	1.404 (4)
C22—C23	1.512 (4)	C442—H442	0.9500
C22—H22A	0.9900	C443—C444	1.381 (4)
C22—H22B	0.9900	C443—H443	0.9500
C23—N24	1.463 (4)	C444—O447	1.375 (4)
C23—H23A	0.9900	C444—C445	1.395 (5)
C23—H23B	0.9900	C445—C446	1.376 (4)
N24—C241	1.419 (3)	C445—H445	0.9500
N24—C25	1.453 (3)	C446—H446	0.9500
C25—C26	1.512 (4)	O447—C447	1.427 (4)
C25—H25A	0.9900	C447—H47A	0.9800
C25—H25B	0.9900	C447—H47B	0.9800
C26—H26A	0.9900	C447—H47C	0.9800
C26—H26B	0.9900	C51—C56	1.387 (4)
C241—C242	1.391 (4)	C51—C52	1.400 (4)
C241—C246	1.394 (4)	C51—C57	1.522 (3)
C242—C243	1.381 (4)	C52—C53	1.396 (4)
C242—H242	0.9500	C52—C58	1.523 (4)
C243—C244	1.395 (4)	C53—C54	1.389 (4)
C243—H243	0.9500	C53—H53	0.9500
C244—O244	1.374 (3)	C54—C55	1.384 (5)
C244—C245	1.380 (4)	C54—H54	0.9500
C245—C246	1.398 (4)	C55—C56	1.389 (4)
C245—H245	0.9500	C55—H55	0.9500
C246—H246	0.9500	C56—H56	0.9500
O244—C247	1.422 (4)	C57—O52	1.246 (3)
C247—H27A	0.9800	C57—O51	1.250 (3)
C247—H27B	0.9800	C58—O54	1.230 (3)
C247—H27C	0.9800	C58—O53	1.267 (4)
N31—C32	1.490 (4)	C61—C66	1.399 (4)
N31—C36	1.491 (4)	C61—C62	1.400 (4)
N31—H31	0.94 (4)	C61—C67	1.511 (4)
N31—H32	0.92 (4)	C62—C63	1.388 (4)
C32—C33	1.515 (4)	C62—C68	1.511 (4)
C32—H32A	0.9900	C63—C64	1.380 (4)
C32—H32B	0.9900	C63—H63	0.9500
C33—N34	1.463 (3)	C64—C65	1.388 (4)
C33—H33A	0.9900	C64—H64	0.9500
C33—H33B	0.9900	C65—C66	1.393 (4)
N34—C341	1.416 (3)	C65—H65	0.9500
N34—C35	1.468 (3)	C66—H66	0.9500
C35—C36	1.511 (4)	C67—O61	1.255 (3)
C35—H35A	0.9900	C67—O62	1.255 (3)
C35—H35B	0.9900	C68—O64	1.229 (3)
C36—H36A	0.9900	C68—O63	1.281 (3)
			
C16—N11—C12	111.8 (2)	N34—C35—H35B	109.5
C16—N11—H11	111 (2)	C36—C35—H35B	109.5
C12—N11—H11	110.2 (19)	H35A—C35—H35B	108.1
C16—N11—H12	107 (3)	N31—C36—C35	110.2 (2)
C12—N11—H12	113 (3)	N31—C36—H36A	109.6
H11—N11—H12	104 (3)	C35—C36—H36A	109.6
N11—C12—C13	110.8 (2)	N31—C36—H36B	109.6
N11—C12—H12A	109.5	C35—C36—H36B	109.6
C13—C12—H12A	109.5	H36A—C36—H36B	108.1
N11—C12—H12B	109.5	C346—C341—C342	117.4 (3)
C13—C12—H12B	109.5	C346—C341—N34	119.3 (2)
H12A—C12—H12B	108.1	C342—C341—N34	123.3 (2)
N14—C13—C12	110.6 (2)	C343—C342—C341	121.7 (3)
N14—C13—H13A	109.5	C343—C342—H342	119.1
C12—C13—H13A	109.5	C341—C342—H342	119.1
N14—C13—H13B	109.5	C344—C343—C342	119.6 (3)
C12—C13—H13B	109.5	C344—C343—H343	120.2
H13A—C13—H13B	108.1	C342—C343—H343	120.2
C141—N14—C15	116.9 (2)	C343—C344—O344	123.8 (3)
C141—N14—C13	114.9 (2)	C343—C344—C345	119.0 (3)
C15—N14—C13	110.1 (2)	O344—C344—C345	117.1 (3)
N14—C15—C16	108.6 (2)	C346—C345—C344	121.4 (3)
N14—C15—H15A	110.0	C346—C345—H345	119.3
C16—C15—H15A	110.0	C344—C345—H345	119.3
N14—C15—H15B	110.0	C345—C346—C341	120.8 (3)
C16—C15—H15B	110.0	C345—C346—H346	119.6
H15A—C15—H15B	108.4	C341—C346—H346	119.6
N11—C16—C15	110.8 (2)	C344—O344—C347	116.8 (2)
N11—C16—H16A	109.5	O344—C347—H37A	109.5
C15—C16—H16A	109.5	O344—C347—H37B	109.5
N11—C16—H16B	109.5	H37A—C347—H37B	109.5
C15—C16—H16B	109.5	O344—C347—H37C	109.5
H16A—C16—H16B	108.1	H37A—C347—H37C	109.5
C146—C141—C142	117.7 (2)	H37B—C347—H37C	109.5
C146—C141—N14	124.4 (2)	C42—N41—C46	111.7 (2)
C142—C141—N14	117.8 (2)	C42—N41—H41	105 (2)
C143—C142—C141	120.8 (3)	C46—N41—H41	110 (2)
C143—C142—H142	119.6	C42—N41—H42	109 (2)
C141—C142—H142	119.6	C46—N41—H42	111 (2)
C142—C143—C144	121.0 (3)	H41—N41—H42	109 (3)
C142—C143—H143	119.5	N41—C42—C43	111.4 (2)
C144—C143—H143	119.5	N41—C42—H42A	109.3
O144—C144—C145	125.3 (3)	C43—C42—H42A	109.3
O144—C144—C143	115.8 (3)	N41—C42—H42B	109.3
C145—C144—C143	118.8 (3)	C43—C42—H42B	109.3
C144—C145—C146	120.2 (3)	H42A—C42—H42B	108.0
C144—C145—H145	119.9	N44—C43—C42	110.2 (2)
C146—C145—H145	119.9	N44—C43—H43A	109.6
C141—C146—C145	121.5 (3)	C42—C43—H43A	109.6
C141—C146—H146	119.3	N44—C43—H43B	109.6
C145—C146—H146	119.3	C42—C43—H43B	109.6
C144—O144—C147	116.4 (2)	H43A—C43—H43B	108.1
O144—C147—H17A	109.5	C441—N44—C43	117.1 (2)
O144—C147—H17B	109.5	C441—N44—C45	116.1 (2)
H17A—C147—H17B	109.5	C43—N44—C45	110.1 (2)
O144—C147—H17C	109.5	N44—C45—C46	110.4 (2)
H17A—C147—H17C	109.5	N44—C45—H45A	109.6
H17B—C147—H17C	109.5	C46—C45—H45A	109.6
C26—N21—C22	111.6 (2)	N44—C45—H45B	109.6
C26—N21—H21	112 (2)	C46—C45—H45B	109.6
C22—N21—H21	111 (2)	H45A—C45—H45B	108.1
C26—N21—H22	107 (2)	N41—C46—C45	110.8 (2)
C22—N21—H22	111 (2)	N41—C46—H46A	109.5
H21—N21—H22	104 (3)	C45—C46—H46A	109.5
N21—C22—C23	111.0 (2)	N41—C46—H46B	109.5
N21—C22—H22A	109.4	C45—C46—H46B	109.5
C23—C22—H22A	109.4	H46A—C46—H46B	108.1
N21—C22—H22B	109.4	C442—C441—C446	117.2 (3)
C23—C22—H22B	109.4	C442—C441—N44	123.8 (3)
H22A—C22—H22B	108.0	C446—C441—N44	118.8 (3)
N24—C23—C22	110.7 (2)	C441—C442—C443	121.7 (3)
N24—C23—H23A	109.5	C441—C442—H442	119.1
C22—C23—H23A	109.5	C443—C442—H442	119.1
N24—C23—H23B	109.5	C444—C443—C442	119.9 (3)
C22—C23—H23B	109.5	C444—C443—H443	120.0
H23A—C23—H23B	108.1	C442—C443—H443	120.0
C241—N24—C25	117.6 (2)	O447—C444—C443	125.8 (3)
C241—N24—C23	115.1 (2)	O447—C444—C445	115.4 (3)
C25—N24—C23	110.0 (2)	C443—C444—C445	118.9 (3)
N24—C25—C26	108.7 (2)	C446—C445—C444	120.6 (3)
N24—C25—H25A	109.9	C446—C445—H445	119.7
C26—C25—H25A	109.9	C444—C445—H445	119.7
N24—C25—H25B	109.9	C445—C446—C441	121.5 (3)
C26—C25—H25B	109.9	C445—C446—H446	119.2
H25A—C25—H25B	108.3	C441—C446—H446	119.2
N21—C26—C25	110.4 (2)	C444—O447—C447	117.0 (2)
N21—C26—H26A	109.6	O447—C447—H47A	109.5
C25—C26—H26A	109.6	O447—C447—H47B	109.5
N21—C26—H26B	109.6	H47A—C447—H47B	109.5
C25—C26—H26B	109.6	O447—C447—H47C	109.5
H26A—C26—H26B	108.1	H47A—C447—H47C	109.5
C242—C241—C246	117.6 (2)	H47B—C447—H47C	109.5
C242—C241—N24	118.2 (2)	C56—C51—C52	119.4 (2)
C246—C241—N24	124.1 (2)	C56—C51—C57	119.0 (2)
C243—C242—C241	121.5 (3)	C52—C51—C57	121.6 (2)
C243—C242—H242	119.2	C53—C52—C51	119.0 (3)
C241—C242—H242	119.2	C53—C52—C58	117.6 (2)
C242—C243—C244	120.4 (3)	C51—C52—C58	123.2 (2)
C242—C243—H243	119.8	C54—C53—C52	121.0 (3)
C244—C243—H243	119.8	C54—C53—H53	119.5
O244—C244—C245	125.0 (3)	C52—C53—H53	119.5
O244—C244—C243	115.9 (3)	C55—C54—C53	119.7 (3)
C245—C244—C243	119.1 (3)	C55—C54—H54	120.1
C244—C245—C246	120.1 (3)	C53—C54—H54	120.1
C244—C245—H245	120.0	C54—C55—C56	119.6 (3)
C246—C245—H245	120.0	C54—C55—H55	120.2
C241—C246—C245	121.3 (3)	C56—C55—H55	120.2
C241—C246—H246	119.4	C51—C56—C55	121.2 (3)
C245—C246—H246	119.4	C51—C56—H56	119.4
C244—O244—C247	117.3 (3)	C55—C56—H56	119.4
O244—C247—H27A	109.5	O52—C57—O51	124.9 (2)
O244—C247—H27B	109.5	O52—C57—C51	118.0 (2)
H27A—C247—H27B	109.5	O51—C57—C51	117.1 (2)
O244—C247—H27C	109.5	O54—C58—O53	125.9 (3)
H27A—C247—H27C	109.5	O54—C58—C52	119.1 (3)
H27B—C247—H27C	109.5	O53—C58—C52	114.8 (2)
C32—N31—C36	111.5 (2)	C66—C61—C62	119.1 (2)
C32—N31—H31	104 (2)	C66—C61—C67	118.8 (2)
C36—N31—H31	111 (2)	C62—C61—C67	122.0 (2)
C32—N31—H32	108 (2)	C63—C62—C61	119.5 (2)
C36—N31—H32	113 (2)	C63—C62—C68	117.4 (2)
H31—N31—H32	109 (3)	C61—C62—C68	122.8 (2)
N31—C32—C33	111.2 (2)	C64—C63—C62	121.1 (3)
N31—C32—H32A	109.4	C64—C63—H63	119.4
C33—C32—H32A	109.4	C62—C63—H63	119.4
N31—C32—H32B	109.4	C63—C64—C65	119.9 (3)
C33—C32—H32B	109.4	C63—C64—H64	120.0
H32A—C32—H32B	108.0	C65—C64—H64	120.0
N34—C33—C32	110.0 (2)	C64—C65—C66	119.6 (3)
N34—C33—H33A	109.7	C64—C65—H65	120.2
C32—C33—H33A	109.7	C66—C65—H65	120.2
N34—C33—H33B	109.7	C65—C66—C61	120.7 (3)
C32—C33—H33B	109.7	C65—C66—H66	119.6
H33A—C33—H33B	108.2	C61—C66—H66	119.6
C341—N34—C33	116.0 (2)	O61—C67—O62	124.1 (2)
C341—N34—C35	114.7 (2)	O61—C67—C61	117.8 (2)
C33—N34—C35	110.1 (2)	O62—C67—C61	118.2 (2)
N34—C35—C36	110.7 (2)	O64—C68—O63	124.7 (3)
N34—C35—H35A	109.5	O64—C68—C62	120.2 (2)
C36—C35—H35A	109.5	O63—C68—C62	114.9 (2)
			
C16—N11—C12—C13	−51.3 (3)	C344—C345—C346—C341	−1.1 (5)
N11—C12—C13—N14	54.6 (3)	C342—C341—C346—C345	−0.3 (5)
C12—C13—N14—C141	164.1 (2)	N34—C341—C346—C345	177.6 (3)
C12—C13—N14—C15	−61.4 (3)	C343—C344—O344—C347	−44.2 (5)
C141—N14—C15—C16	−163.5 (2)	C345—C344—O344—C347	139.9 (3)
C13—N14—C15—C16	63.1 (3)	C46—N41—C42—C43	−52.1 (3)
C12—N11—C16—C15	54.0 (3)	N41—C42—C43—N44	56.6 (3)
N14—C15—C16—N11	−59.3 (3)	C42—C43—N44—C441	163.5 (2)
C15—N14—C141—C146	−12.7 (4)	C42—C43—N44—C45	−61.0 (3)
C13—N14—C141—C146	118.6 (3)	C441—N44—C45—C46	−162.8 (2)
C15—N14—C141—C142	165.0 (2)	C43—N44—C45—C46	61.2 (3)
C13—N14—C141—C142	−63.7 (3)	C42—N41—C46—C45	51.8 (3)
C146—C141—C142—C143	0.2 (4)	N44—C45—C46—N41	−56.2 (3)
N14—C141—C142—C143	−177.6 (3)	C43—N44—C441—C442	6.8 (4)
C141—C142—C143—C144	0.5 (5)	C45—N44—C441—C442	−126.0 (3)
C142—C143—C144—O144	179.6 (3)	C43—N44—C441—C446	−168.4 (3)
C142—C143—C144—C145	−0.6 (5)	C45—N44—C441—C446	58.7 (3)
O144—C144—C145—C146	179.7 (3)	C446—C441—C442—C443	3.4 (4)
C143—C144—C145—C146	0.0 (4)	N44—C441—C442—C443	−171.9 (3)
C142—C141—C146—C145	−0.9 (4)	C441—C442—C443—C444	−1.1 (4)
N14—C141—C146—C145	176.8 (2)	C442—C443—C444—O447	177.8 (3)
C144—C145—C146—C141	0.7 (4)	C442—C443—C444—C445	−2.1 (4)
C145—C144—O144—C147	10.7 (4)	O447—C444—C445—C446	−177.1 (3)
C143—C144—O144—C147	−169.6 (3)	C443—C444—C445—C446	2.8 (5)
C26—N21—C22—C23	−51.3 (3)	C444—C445—C446—C441	−0.4 (5)
N21—C22—C23—N24	54.2 (3)	C442—C441—C446—C445	−2.7 (4)
C22—C23—N24—C241	163.3 (2)	N44—C441—C446—C445	172.9 (3)
C22—C23—N24—C25	−61.0 (3)	C443—C444—O447—C447	0.5 (4)
C241—N24—C25—C26	−162.1 (2)	C445—C444—O447—C447	−179.6 (3)
C23—N24—C25—C26	63.4 (3)	C56—C51—C52—C53	−2.0 (4)
C22—N21—C26—C25	54.4 (3)	C57—C51—C52—C53	174.2 (2)
N24—C25—C26—N21	−60.0 (3)	C56—C51—C52—C58	172.7 (2)
C25—N24—C241—C242	164.0 (3)	C57—C51—C52—C58	−11.1 (4)
C23—N24—C241—C242	−63.8 (3)	C51—C52—C53—C54	0.8 (4)
C25—N24—C241—C246	−12.4 (4)	C58—C52—C53—C54	−174.2 (3)
C23—N24—C241—C246	119.8 (3)	C52—C53—C54—C55	0.9 (4)
C246—C241—C242—C243	0.0 (4)	C53—C54—C55—C56	−1.3 (5)
N24—C241—C242—C243	−176.6 (3)	C52—C51—C56—C55	1.6 (4)
C241—C242—C243—C244	0.2 (5)	C57—C51—C56—C55	−174.8 (3)
C242—C243—C244—O244	179.8 (3)	C54—C55—C56—C51	0.1 (4)
C242—C243—C244—C245	0.0 (5)	C56—C51—C57—O52	141.7 (3)
O244—C244—C245—C246	179.9 (3)	C52—C51—C57—O52	−34.5 (4)
C243—C244—C245—C246	−0.3 (4)	C56—C51—C57—O51	−36.7 (4)
C242—C241—C246—C245	−0.3 (4)	C52—C51—C57—O51	147.0 (3)
N24—C241—C246—C245	176.0 (3)	C53—C52—C58—O54	−60.9 (4)
C244—C245—C246—C241	0.5 (4)	C51—C52—C58—O54	124.3 (3)
C245—C244—O244—C247	2.9 (4)	C53—C52—C58—O53	114.5 (3)
C243—C244—O244—C247	−176.9 (3)	C51—C52—C58—O53	−60.2 (3)
C36—N31—C32—C33	−53.7 (3)	C66—C61—C62—C63	−2.0 (4)
N31—C32—C33—N34	56.5 (3)	C67—C61—C62—C63	174.2 (2)
C32—C33—N34—C341	167.6 (2)	C66—C61—C62—C68	171.6 (2)
C32—C33—N34—C35	−60.0 (3)	C67—C61—C62—C68	−12.3 (4)
C341—N34—C35—C36	−166.1 (2)	C61—C62—C63—C64	0.9 (4)
C33—N34—C35—C36	60.9 (3)	C68—C62—C63—C64	−173.0 (2)
C32—N31—C36—C35	53.6 (3)	C62—C63—C64—C65	0.5 (4)
N34—C35—C36—N31	−57.2 (3)	C63—C64—C65—C66	−0.8 (4)
C33—N34—C341—C346	−173.4 (3)	C64—C65—C66—C61	−0.4 (4)
C35—N34—C341—C346	56.4 (3)	C62—C61—C66—C65	1.7 (4)
C33—N34—C341—C342	4.3 (4)	C67—C61—C66—C65	−174.6 (2)
C35—N34—C341—C342	−125.9 (3)	C66—C61—C67—O61	−34.9 (4)
C346—C341—C342—C343	1.6 (4)	C62—C61—C67—O61	149.0 (3)
N34—C341—C342—C343	−176.1 (3)	C66—C61—C67—O62	144.0 (3)
C341—C342—C343—C344	−1.6 (5)	C62—C61—C67—O62	−32.2 (4)
C342—C343—C344—O344	−175.6 (3)	C63—C62—C68—O64	−64.0 (3)
C342—C343—C344—C345	0.2 (5)	C61—C62—C68—O64	122.3 (3)
C343—C344—C345—C346	1.2 (5)	C63—C62—C68—O63	112.2 (3)
O344—C344—C345—C346	177.3 (3)	C61—C62—C68—O63	−61.4 (3)

### Bis[4-(4-methoxyphenyl)piperazin-1-ium] benzene-1,2-dicarboxylate (II) Hydrogen-bond geometry (Å, º)

*Cg*3 and *Cg*4 represent the centroids of the C61–C66 and C51–C56 rings, respectively.

**Table:**

*D*—H···*A*	*D*—H	H···*A*	*D*···*A*	*D*—H···*A*
N11—H11···O51	0.99 (4)	1.73 (4)	2.714 (4)	175 (2)
N11—H12···O63^i^	0.80 (4)	2.03 (4)	2.738 (3)	148 (3)
N21—H21···O61	0.99 (4)	1.72 (4)	2.707 (4)	177 (3)
N21—H22···O53^ii^	1.01 (4)	1.88 (4)	2.744 (3)	142 (3)
N31—H31···O63^iii^	0.94 (3)	1.77 (3)	2.685 (3)	163 (3)
N31—H32···O52	0.92 (4)	1.83 (4)	2.732 (3)	164 (3)
N41—H41···O53^iv^	1.00 (4)	1.74 (4)	2.711 (3)	163 (3)
N41—H42···O62	0.90 (4)	1.89 (4)	2.740 (3)	157 (3)
C36—H36*A*···O51^v^	0.99	2.40	3.354 (4)	160
C46—H46*A*···O61^v^	0.99	2.42	3.359 (4)	158
C13—H13*B*···*Cg*3^i^	0.99	2.84	3.795 (3)	161
C23—H23*B*···*Cg*4^i^	0.99	2.85	3.800 (3)	162

Symmetry codes: (i) −*x*+3/2, *y*+1/2, *z*−1/2; (ii) −*x*+3/2, *y*−1/2, *z*+1/2; (iii) −*x*+1, −*y*+1, *z*−1/2; (iv) −*x*+1, −*y*+2, *z*+1/2; (v) *x*−1/2, −*y*+3/2, *z*.
